# Impact of a deep learning sepsis prediction model on quality of care and survival

**DOI:** 10.1038/s41746-023-00986-6

**Published:** 2024-01-23

**Authors:** Aaron Boussina, Supreeth P. Shashikumar, Atul Malhotra, Robert L. Owens, Robert El-Kareh, Christopher A. Longhurst, Kimberly Quintero, Allison Donahue, Theodore C. Chan, Shamim Nemati, Gabriel Wardi

**Affiliations:** 1 Department of Medicine, University of California San Diego, San Diego, CA USA; 2 Department of Quality, University of California San Diego, San Diego, CA USA; 3 Department of Emergency Medicine, University of California San Diego, San Diego, CA USA

**Keywords:** Prognosis, Preventive medicine

## Abstract

Sepsis remains a major cause of mortality and morbidity worldwide. Algorithms that assist with the early recognition of sepsis may improve outcomes, but relatively few studies have examined their impact on real-world patient outcomes. Our objective was to assess the impact of a deep-learning model (COMPOSER) for the early prediction of sepsis on patient outcomes. We completed a before-and-after quasi-experimental study at two distinct Emergency Departments (EDs) within the UC San Diego Health System. We included 6217 adult septic patients from 1/1/2021 through 4/30/2023. The exposure tested was a nurse-facing Best Practice Advisory (BPA) triggered by COMPOSER. In-hospital mortality, sepsis bundle compliance, 72-h change in sequential organ failure assessment (SOFA) score following sepsis onset, ICU-free days, and the number of ICU encounters were evaluated in the pre-intervention period (705 days) and the post-intervention period (145 days). The causal impact analysis was performed using a Bayesian structural time-series approach with confounder adjustments to assess the significance of the exposure at the 95% confidence level. The deployment of COMPOSER was significantly associated with a 1.9% absolute reduction (17% relative decrease) in in-hospital sepsis mortality (95% CI, 0.3%–3.5%), a 5.0% absolute increase (10% relative increase) in sepsis bundle compliance (95% CI, 2.4%–8.0%), and a 4% (95% CI, 1.1%–7.1%) reduction in 72-h SOFA change after sepsis onset in causal inference analysis. This study suggests that the deployment of COMPOSER for early prediction of sepsis was associated with a significant reduction in mortality and a significant increase in sepsis bundle compliance.

## Introduction

Sepsis, a dysregulated host response to infection, is estimated to afflict over 48.9 million people a year worldwide of whom approximately 11 million die^1,2^. The early recognition of sepsis is critical since interventions such as fluid resuscitation, antibiotic administration, and source control all have greater benefits when implemented earlier in the disease course^3–9^. The detection of patients with sepsis can be challenging due to the heterogeneity of the condition; thus, we and others have used predictive analytics to improve the early detection^10–14^. We recently reported the performance of COMPOSER, a deep-learning model that imports real-time data from electronic health records to predict sepsis before obvious clinical manifestations^15^. Few sepsis algorithms have been rigorously tested at the bedside or evaluated with regard to patient outcomes^16–19^. Existing algorithms within electronic health records (EHRs) have demonstrated relatively poor positive predictive value (PPV) and may contribute to provider mistrust of predictive models^20,21^. Of note, false positive alerts from such models often lead to alarm fatigue and provider burnout/mistrust. COMPOSER was specifically designed to reduce false alarms by flagging outliers and out-of-distribution samples as indeterminate^15^.

Based on this conceptual framework, we integrated the COMPOSER algorithm in two emergency departments (ED) at UC San Diego (UCSD) Health via our EHR (Epic Systems, Verona, WI). We seek to test the hypothesis that our algorithm-based intervention was feasible in real-time and that the additional information would help to guide clinicians to earlier sepsis recognition and result in improved patient outcomes. To accomplish this goal, we conduct a quasi-experimental study in which we track outcomes before and after deployment, with historical control data to account for baseline acuity, comorbidities, seasonal effects, and secular trends over time.

## Results

During the study period, January 1st, 2021 through April 30th, 2023, 6,340 ED encounters met the Sepsis-3 consensus sepsis definition, of which 123 were excluded because they were transitioned to comfort measures before sepsis onset. The final study included 6217 patients, 5065 in the pre-intervention phase, and 1152 in the post-intervention phase. Table 1 shows baseline characteristics and summary statistics for the study cohort. Baseline characteristics from each emergency department are compared in Supplementary Table 1. Most septic patients exhibited some level of chronic comorbidity (median Elixhauser of 5) and the median SOFA score at the time of sepsis was 2. We did not observe significant differences in the baseline characteristics between the pre-intervention cases and post-intervention cases.

**Table 1:** Demographics and baseline characteristics of septic patients before and after COMPOSER.

	Total	Pre-intervention	Post-intervention	*P-*value^a^
Characteristic				
Number of patients, *N* (%)	6217 (100%)	5065 (81.5%)	1152 (18.5%)	-
Age, mean (SD)	63 (17.1)	63 (17.0)	64 (17.3)	0.08
Sex, *N* (%)				
Male	3592 (57.8%)	2966 (58.6%)	626 (54.3%)	-
Female	2625 (42.2%)	2099 (41.4%)	526 (45.7%)	-
Race				
Asian	530 (8.5%)	404 (8%)	126 (10.9%)	-
Black or African American	639 (10.3%)	519 (10.2%)	120 (10.4%)	-
White	2983 (48%)	2440 (48.2%)	543 (47.1%)	-
Other^b^	2065 (33.2%)	1702 (33.6%)	363 (31.5%)	-
Ethnic group				-
Hispanic/Latino	1756 (28.2%)	1449 (28.6%)	307 (26.6%)	-
Not Hispanic/Latino	4461 (71.8%)	3616 (71.4%)	845 (73.4%)	-
Organ dysfunction				
Elixhauser Comorbidity Index, Median (IQR)	5 (0–13)	5 (0–13)	5 (0–14)	0.64
SOFA Score at Time of Sepsis, Median (IQR)	2 (1–3)	2 (1–3)	2 (1–3)	0.99
Lab values				
Lactate at the time of sepsis	2.4 (1.6–4.3)	2.4 (1.6–4.3)	2.4 (1.6–4.3)	0.76
Interventions				
Mechanical Ventilation, *N* (%)^c^	1035 (16.6%)	849 (16.8%)	186 (16.1%)	0.64
Administration of Vasoactive Medications, *N* (%)^c^	424 (6.8%)	345 (6.8%)	79 (6.9%)	1.0

^a^*P*-values for continuous variables are based on Kruskal–Wallis rank sum tests. *P*-values for categorical variables are based on Pearson’s chi-squared tests.

^b^Other race corresponds to Native Hawaiian or Other Pacific Islander, American Indian or Alaska Native, Other Race or Mixed Race, or Unknown.

^c^Within 72-h of ED arrival.

### COMPOSER alerts

**Fig. 1:**
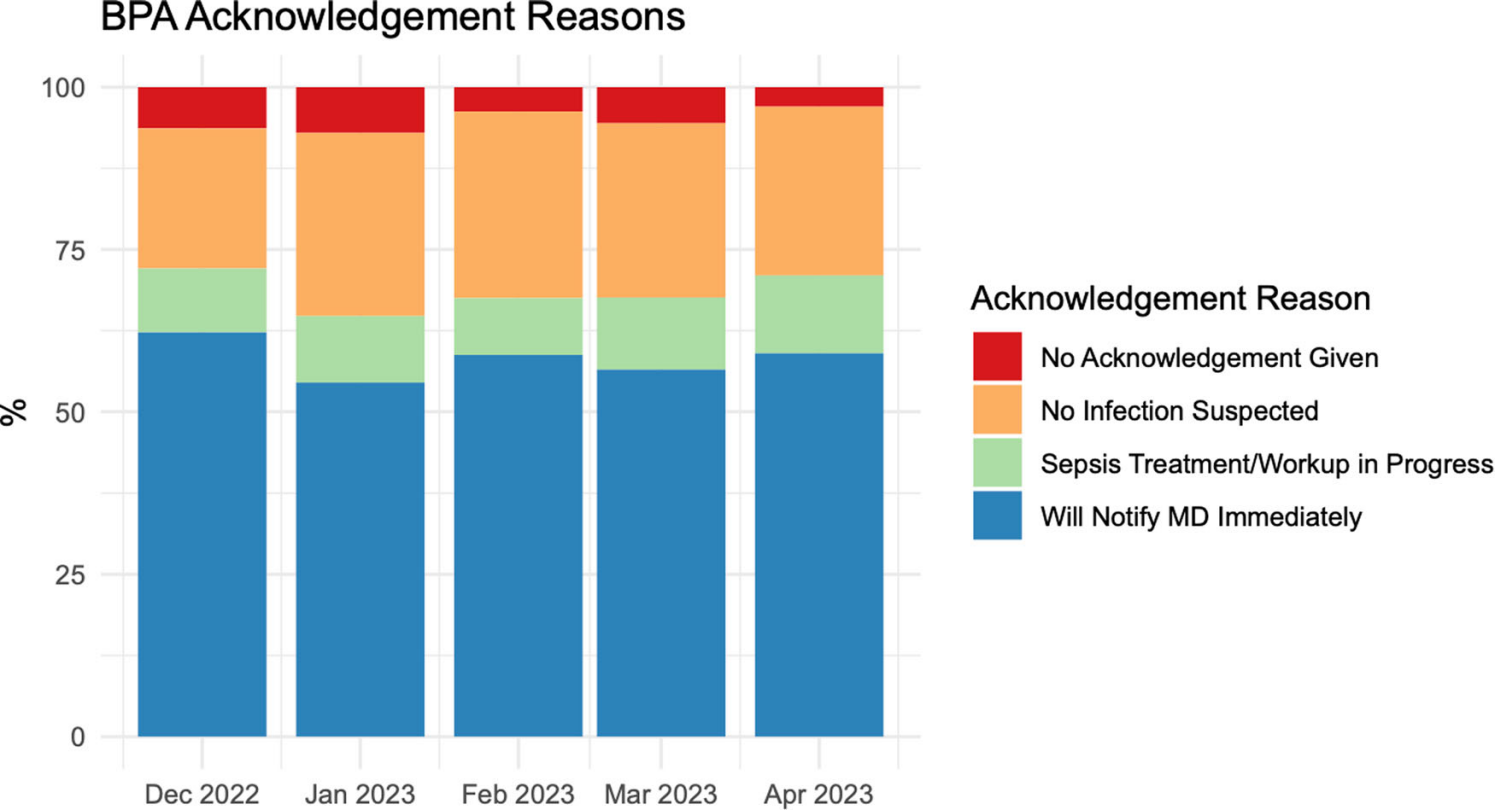
Acknowledgements to Each COMPOSER Best Practice Advisory alert from December, 2022 until April, 2023.

During the post-intervention period, an average of 235 alerts were generated per month corresponding to 1.65 alerts per nurse per month. Alerts by acknowledgement reason are visualized in Fig. 1. The most common acknowledgement reason was “Will Notify MD Immediately” which comprised over half of all acknowledgement reasons. Only about 5.9% of BPAs were exited without acknowledgement and responses to the BPA remained consistent across the 5-month intervention period.

### Interventions and patient outcomes

**Fig. 2:**
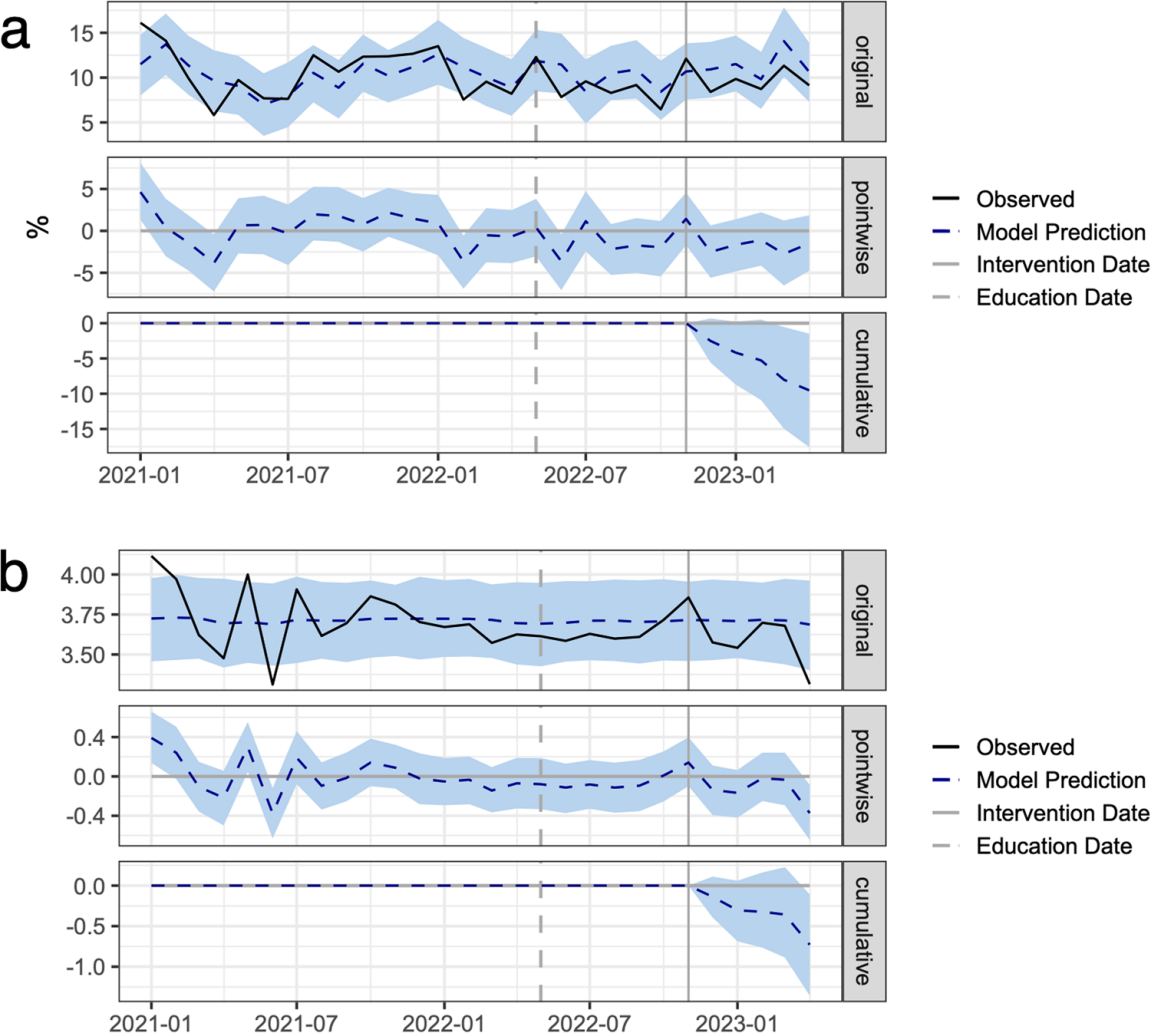
Causal impact analysis of COMPOSER Best Practice Advisory on patient outcomes. Plots of the causal impact analysis using a Bayesian structural time-series model. The top subpanel (“original”) shows the actual outcome (black) and the average model predictions (dashed blue) and 95% confidence limits (shaded blue) during the pre-intervention and post-intervention periods, indicated by the solid gray vertical line. The middle subpanel (“pointwise”) shows the difference between the model predictions and the observed outcome. The bottom subpanel (“cumulative”) shows the sum of the pointwise differences during the post-intervention period. Preparation for the implementation of COMPOSER began in May 2022 approximately 6 months prior to the go-live date of the model. **a** The cumulative post-intervention in-hospital sepsis mortality rate is below the 95% confidence limit. **b** The cumulative post-intervention 72-h change in SOFA score is below the 95% confidence limit.

The results from causal impact analysis on our primary and secondary outcomes are summarized in Table 2. The observed in-hospital mortality rate and the corresponding predictions from the Bayesian structural time-series model are shown in Fig. 2a. The residual quantile-quantile and autocorrelation plots are described in Supplementary Figs. 4 and 5. The average sepsis mortality rate during the post-intervention period was 9.49%. If the COMPOSER algorithm had not been deployed, the expected counterfactual mortality rate would have been 11.39% with a 95% confidence interval of [9.79%, 13.00%], corresponding to a 1.9% absolute decrease in sepsis-related in-hospital mortality. This value corresponds to a 17% relative decrease in in-hospital mortality among patients with sepsis and 22 additional patients who survived during the 5-month intervention period. The probability of this occurring by chance is determined from the Bayesian one-sided tail-area probability, *p* = 0.014. Additional data regarding the difference in mortality at our two hospitals are provided in Supplementary Figs. 6–9. We found at one site (the “safety net” hospital) we had a significant decrease in mortality in the post-intervention period, but we did not observe a significant change at the other clinical site (the quaternary care facility).

**Table 2:** Observed outcomes in the pre-intervention period, the expected counterfactual values from causal impact analysis, and the actual post-intervention values.

Outcome	Pre-intervention value	Expected post-intervention value (95% CI)	Actual post-intervention value
In-hospital mortality %	10.3%	11.4% (9.8%−13.0%)	**9.5%**
Average 72-h Change in SOFA	3.71	3.71 (3.6–3.8)	**3.56**
Sepsis bundle compliance rate	48.3%	48.4% (45.5%−51.0%)	**53.4%**
Blood cultures prior to antibiotics compliance rate	71.1%	72.0% (69.9%−73.9%)	73.9%
Rate of antibiotics administered within 24 h prior and 3 h after severe sepsis onset.	82.8%	82.8% (81.3%−84.4%)	**84.6%**
Rate of lactate measured within 6 h prior and 3 h after severe sepsis onset	83.5%	83.4% (81.3%−85.8%)	85.6%
Rate of repeat lactate measured within 6 h after severe sepsis onset if initial lactate is elevated	97.8%	97.3% (96.2%−98.4%)	**98.6%**
Rate of administration of vasoactive medications within 6 h of septic shock	58.0%	57.5% (46.7%−68.2%)	55.5%
Rate of administration of 30cc/kg of fluids within 3 h of presentation of septic shock or hypotension	54.2%	53.9% (48.9%−58.8%)	**59.3%**
ICU transfer rate	32.6%	32.5% (30.7%−34.2%)	31.8%
Average ICU-free days	25.4	25.1 (24.6–25.6)	25.6

Significant post-intervention values against the 95% confidence interval are bolded.

**Fig. 3:**
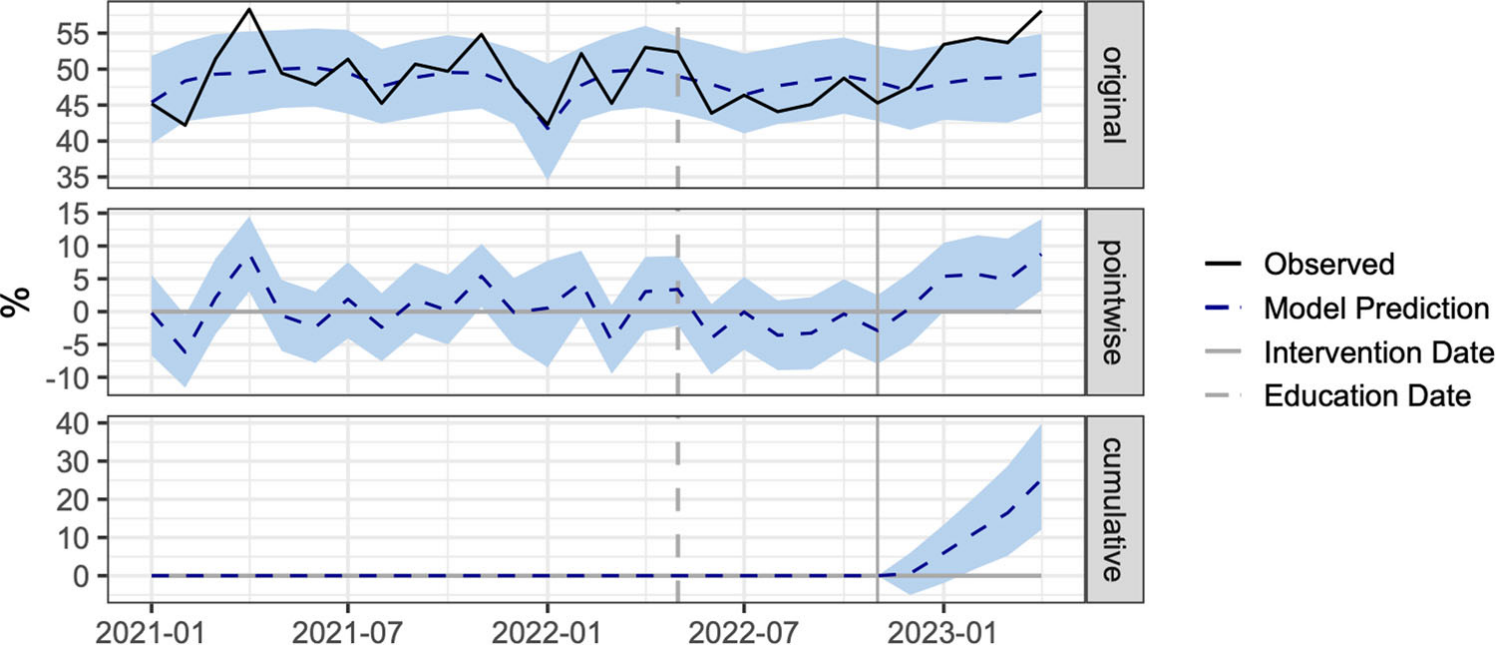
Causal impact analysis of COMPOSER Best Practice Advisory (BPA) on sepsis bundle compliance rate. Implementation of the COMPOSER BPA was significantly associated with an increase in sepsis bundle compliance.

The average compliance rate during the post-intervention period was 53.42% while, in the absence of the COMPOSER intervention, the expected compliance rate would have been 48.38% (95% CI, 45.46%–51.01%; Fig. 3), or a 5.0% (95% CI, 2.4%–8.0%) increase in compliance. This corresponds to a 10% (95% CI, 5%–16%) relative increase in sepsis bundle compliance following the implementation of COMPOSER. As shown in Supplementary Figs. 10 and 11, compliance with our sepsis bundle increased at both EDs during the intervention period. Compliance with specific bundle elements is shown in Table 2 and Supplementary Figs. 12–17. We observe significant improvements in antibiotic compliance, repeat lactate compliance, and administration of fluids compliance.

We also observed a reduction in the 72-h change in SOFA score following sepsis onset (Fig. 2b). The average change in SOFA score during the post-intervention period was 3.56. In the absence of the COMPOSER intervention, however, the expected counterfactual average change in SOFA score would have been about 3.71 with a 95% confidence interval of [3.58, 3.83]. This corresponds to a 4% decrease in the average 72-h change in SOFA score following sepsis onset. The probability of this occurring by chance is *p* = 0.013. Additional data on the change in SOFA score at each emergency department are provided in Supplementary Figs. 18 and 19. We further observe a downward trend in our secondary endpoint of ICU admissions (Supplementary Fig. 20) and an upward trend in ICU-free days (Supplementary Fig. 21) although neither reaches statistical significance. The temporal trends of all covariates used in the Bayesian structural time-series models are provided in Supplementary Fig. 22.

Associations between time-to-antibiotics in septic patients and acknowledgement reasons are shown in Table 3. We observe that in cases where nurses indicated that they would notify physicians immediately, there was a significant reduction in time to antibiotic administration (*p* = 0.002; two-sided *t*-test with adjustments for ED volume, sex, baseline SOFA, Elixhauser comorbidity score, and age).

**Table 3:** Associations Between BPA Response and Antibiotics Timing.

Variable	Coefficient [95% CI]	*P* Value^a^
(Intercept)	22.42 [−45.27–90.11]	0.513
Age	0.01 [−0.15–0.18]	0.875
Male	−0.53 [−5.99–4.93]	0.848
Elixhauser Comorbidity Index	0.21 [−0.10–0.52]	0.180
Baseline SOFA	1.12 [−2.89–5.13]	0.581
Monthly ED Volume (in thousands)	0.62 [−13.33–14.58]	0.930
BPA Acknowledgement: No Infection Suspected	−13.24 [−26.60–0.127]	0.052
BPA Acknowledgement: Sepsis Treatment/Workup in Progress	−19.16 [−33.73–−4.60]	0.010
BPA Acknowledgement: Will Notify MD Immediately	−19.95 [−32.39–−7.51]	0.002

^a^*P* values are based on two-sided t-tests with adjustments for ED volume, sex, baseline SOFA, Elixhauser comorbidity score, and age.

Coefficients and *P* values are tabulated for a linear regression model with adjustments. If the nurse selected “No Infection Suspected” on an 80-year-old male with an Elixhauser of 4 and a baseline SOFA of 1, the expected “time from ED triage to antibiotics administration” would be 14.5 hours. However, if the nurse had selected “Will Notify MD Immediately” the expected “time from ED triage to antibiotics administration” would have been 7.7 hours.

## Discussion

In this before-and-after quasi-experimental study, we demonstrated that the implementation of a real-time deep-learning model to predict sepsis in two EDs was associated with a 5.0% absolute increase in sepsis bundle compliance and a 1.9% absolute decrease in-hospital sepsis-related mortality. This finding represents, to our knowledge, the first instance of prospective use of a deep-learning model demonstrating an association with improved patient-centered outcomes in sepsis. Our findings also suggest that the utilization of such models in clinical care was also associated with improvements in intermediate outcomes, such as less organ injury at 72 h from the time of sepsis and improvements in elements of sepsis bundles which may explain the mortality benefit described. Importantly, we show in scenarios where nursing staff reported notification of the provider with concern for sepsis (approximately 55% of cases) that antibiotics were administered sooner, providing a plausible mechanism for the lower-than-expected in-hospital mortality we report.

Despite major interest in strategies to relieve the morbidity and mortality of sepsis, novel therapeutics have failed to translate into meaningful patient-centered outcomes. The potential to improve care through the use of artificial intelligence is attractive, particularly with advances in machine learning in the past decade^22,23^. Unfortunately, the majority of algorithms designed to predict sepsis never make it to the bedside^24^. Older models designed to detect sepsis were largely based on clinical criteria (i.e., SIRS criteria, hypotension, or a combination of these). These models were associated with occasional improvement in quality metrics (i.e., increased rates of lactate orders or time to antibiotics), but did not improve patient-centered outcomes and had poor PPV^25–27^.

More recently, several studies have implemented sophisticated models at various hospitals showing benefits to patients. Shimaburuko et al. conducted a small randomized trial of 142 patients in the ICU using a machine-learning algorithm to predict severe sepsis and found a decrease in in-hospital mortality and length of stay in the intervention group, although this study was limited to patients either in the hospital wards or intensive care units^18^. Adams et al. recently provided a prospective analysis of the TREWS model at five hospital systems in which they demonstrated a significant decrease in mortality, organ failure, and length of stay in hospitalized patients when the sepsis alert was confirmed by a provider^16^. While this study was not randomized, the data are compelling that proper attention to implementation may improve patient-centered outcomes in sepsis.

However, a commonly used predictive model, the Epic Sepsis Score (ESS), has not demonstrated consistent improvement in patient-centered outcomes. Although a small randomized quality improvement initiative from a single center found an improvement of the composite clinical outcome measure of days alive and out of hospital at 28 days was greater in the ESS care group, these results have not been generalized thus far. Importantly, researchers at the University of Michigan highlighted a substantial drop in test characteristics (sensitivity, specificity, PPV) of the ESS at their institution from what was reported by Epic, as well as an unacceptably high rate of false positives^20^.

To the best of our knowledge, the only deep-learning model previously tested in an ED setting is the Sepsis Watch by investigators at Duke; however, no patient-centered outcomes have been reported thus far^28^. As such, the present study is the first reporting of improvement in patient-centered outcomes attributable to the deployment of a deep-learning-based sepsis prediction model.

The use of deep learning for early prediction of sepsis is significant since such models are capable of modeling temporal, nonlinear, and complex correlations among risk factors, thus enabling them to solve more difficult problems. Moreover, deep-learning models are capable of handling large quantities of multimodal data from radiology imaging, clinical notes, and wearable sensors, among other^29–32^. Additionally, this class of models provides a flexible framework for transfer learning and continual learning to enable the adoption of such models to local healthcare settings^33–35^.

Importantly, the COMPOSER deep-learning model was designed to minimize false alarms via the conformal prediction framework. This approach imposes a boundary around the algorithm, which enables the model to identify whether it has enough prior knowledge of similar cases to determine reliably whether a patient is at risk for sepsis. If the algorithm finds the data non-conformant to the training samples, it will flag the case as ‘indeterminate’. The resulting reduction in false alarms, previously reported to be 75%, greatly reduces the burden of resources or time spent on false diagnoses.

There are various potential reasons that may explain the reduction in mortality described above. First, we noted a high percentage (~55%) of alerts were transmitted by nursing staff to physicians. In this scenario, we found that these patients were more likely to receive timely antibiotics, thus providing a potential mechanism to decrease mortality and mitigate organ dysfunction at 72 h. The use of artificial intelligence to facilitate a shared mental model of risk between nursing staff and providers has demonstrated good acceptance and improved use of these models in other clinical areas^36^. In our system, for instance, we chose to have the nurses receive the alert and determine if escalation to the provider was appropriate. While the ideal target population for such an intervention is unclear, we felt that our nurses would be the ideal candidate for this alert because of the high frequency of nurses opening patients’ charts. In the author’s collective experience, physicians in the ED may have up to 15–20 patients at a time and may not receive a BPA that requires a chart to be open to receive the notification. Given the high rate of provider notification, we suspect that this approach was beneficial to patient care while additionally minimizing unnecessary alerts. Finally, although speculative, it is possible that the implementation of the alert improved situational awareness of sepsis care within our ED staff. This finding has been reported in other sepsis clinical decision tools as well^37^.

Despite our study’s strengths, we acknowledge several limitations. First, our study was not randomized and thus our findings do not allow definitive causal inferences or mechanistic insights. We performed a causal impact analysis with common confounders which revealed that the implementation was significantly associated with positive outcomes. Regardless, we view the findings as important and believe that they provide a strong rationale for further research. Second, our study was conducted at two EDs in a large academic center that has a major interest in sepsis and clinical informatics. Although we had a large sample size and a diverse population of patients (racial, ethnic, socioeconomic status, etc.), we acknowledge the need for external validation in other healthcare settings (e.g., community hospitals, different demographics, hospitals without robust IT infrastructure, etc.). Third, one could argue that an abrupt intervention has important immediate benefits raising awareness and helping to prioritize the care of a specific group of patients. Conversely, the sustainability of the intervention could be questioned, emphasizing the need for longer-term follow-up. Although human interventions are subject to fatigue and complacency, we anticipate our automated algorithms will improve over time with increasing experience and larger data sets, which will likely result in improvements in end-user satisfaction. However, we certainly recognize the importance of continuous education as a component of care optimization. Finally, we did not evaluate the impact of this alert on patients who ultimately did not have sepsis, such as the potential adverse effects of inappropriate use of antibiotics and healthcare costs associated with this. We also acknowledge that we did not have any comparison data from the same time period as all of our EDs used this model. However, we did not have any other quality improvement initiatives during the same time period. Despite these limitations, we view our new findings as actionable and important.

In the before-and-after quasi-experimental design study conducted at two EDs, we demonstrate that the implementation of a real-time deep-learning model to predict sepsis was associated with a significant increase in bundle compliance, a significant reduction in in-hospital mortality, less organ dysfunction at 72 h, and improved timeliness to antibiotics when nurses notified the physician of the BPA. To our knowledge, this is the first time that the improvement of patient outcomes due to the use of a deep-learning model for sepsis prediction has been reported. Future multicenter randomized trials are indicated to validate these findings across a diverse hospital and patient population.

## Methods

### Study design and cohort

**Fig. 4:**
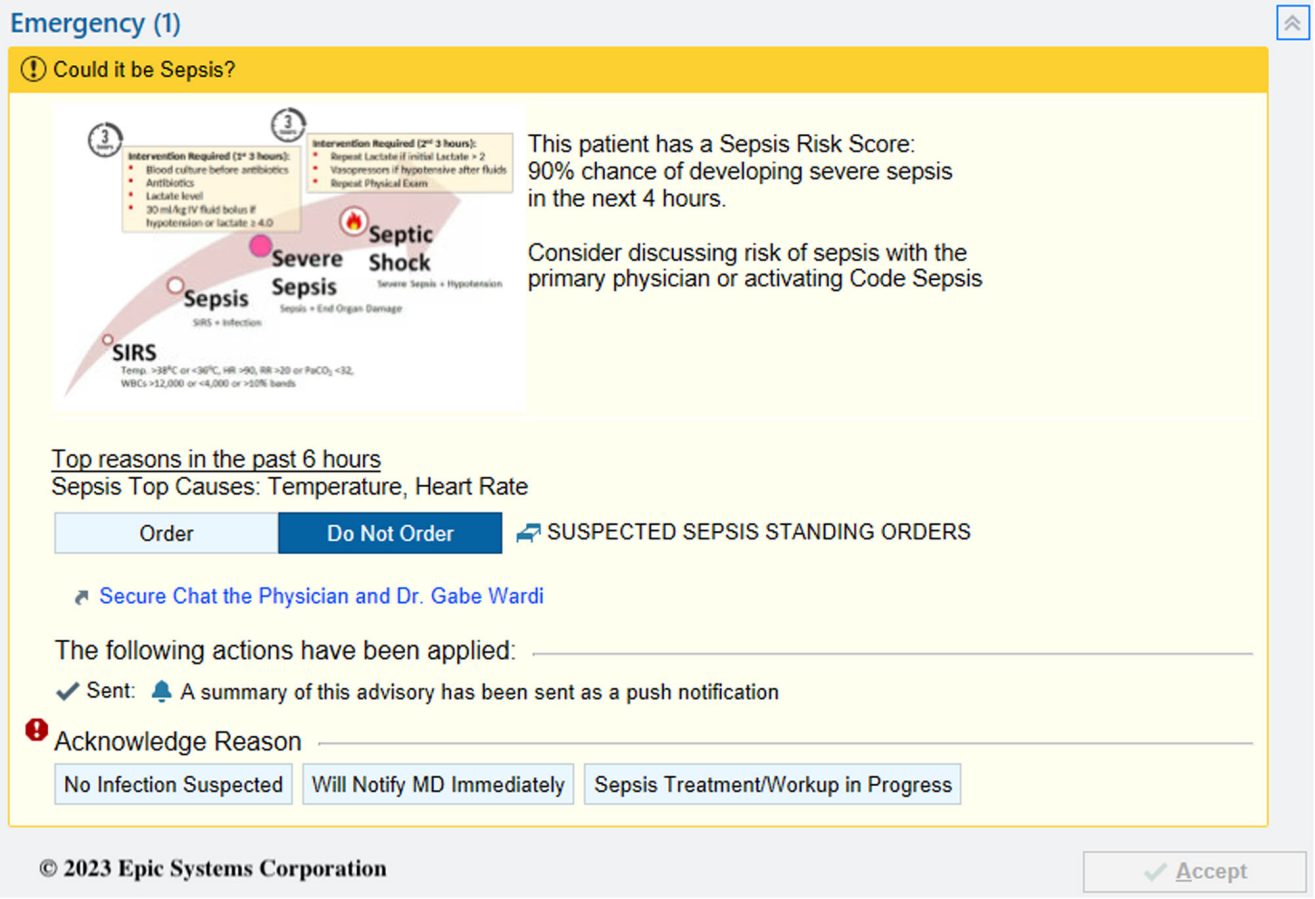
COMPOSER Best Practice Advisory.

We conducted a prospective before-and-after quasi-experimental study to evaluate the impact of a sepsis Best Practice Advisory (BPA; Fig. 4) powered by the COMPOSER deep-learning model on patient outcomes and process measures. The University of California San Diego Institutional review board (IRB) approval was obtained with the waiver of informed consent (#805726) and additional approval was obtained from the Aligning and Coordinating QUality Improvement, Research, and Evaluation (ACQUIRE) Committee (project #609). Our study was completed in accordance with STROBE guidelines^38^. A completed checklist is provided in Supplementary Note 1. These EDs have a total volume of approximately 100,000 patients annually with one serving at a quaternary academic center and the other at an urban “safety net” hospital.

Patients were identified as septic according to the latest international consensus definitions for sepsis (“Sepsis-3”)^1,3^. The onset time of sepsis was established by following previously published methodology, using evidence of organ dysfunction and suspicion of clinical infection^1,12,15^. Clinical suspicion of infection was defined by a blood culture draw and at least 4 days of non-prophylactic intravenous antibiotic therapy satisfying either of the following conditions: (1) if a blood culture draw was ordered first, then an antibiotics order had to occur within the following 72 h, or (2) if an antibiotics order occurred first, then a blood culture draw had to occur within the next 24 h. Evidence of organ dysfunction was defined as an increase in the Sequential Organ Failure Assessment (SOFA) score by two or more points. In particular, evidence of organ dysfunction occurring 48 h before to 24 h after the time of suspected infection was considered, as suggested in Seymour et al.^1^. Finally, the time of onset of sepsis was taken as the time of clinical suspicion of infection. The inclusion of 4 days of non-prophylactic antibiotics to improve the specificity of sepsis is similar to what Rhee et al. proposed as a surveillance approach for identifying sepsis from electronic health records which have outperformed reliance on administrative coding of sepsis^39^. We included all adult patients (age ≥18 years old) who met the criteria for the above-described Sepsis-3 definition within the first 12 h of their ED stay. We excluded patients who were transitioned to comfort measures prior to their time of sepsis and patients who developed sepsis after 12 h of hospital admission. All data used to derive the onset time of sepsis and patient outcomes were extracted via SQL queries on Epic Clarity.

### Sepsis algorithm and platform

The COMPOSER algorithm for the early prediction of sepsis is described in Shashikumar et al.^15^. It is a feed-forward neural network model that incorporates routinely collected laboratory and vital signs as well as patient demographics (age and sex), comorbidities, and concomitant medications to output a risk score for the onset of sepsis within the next 4 h. Importantly, the model utilizes the conformal prediction method to reject out-of-distribution samples that may arise due to data entry error or unfamiliar cases. The model achieves an area under the receiver operating characteristic curve (AUROC) of 0.938–0.945 within ED settings^15^. We fixed the score threshold to achieve an 80% sensitivity level. Prior work demonstrated that at this sensitivity, the PPV was 20.1%.

The COMPOSER algorithm is hosted on a cloud-based healthcare analytics platform that enables access to data elements in real-time by leveraging the FHIR and HL7v2 standards (Supplementary Fig. 1)^40^. Specifically, the Amazon Web Services (AWS)-hosted infrastructure receives a continuous stream of Admit, Discharge, Transfer (ADT) messages from the hospital’s integration engine to determine the active patients and map their journey through care units. The platform extracts data at an hourly resolution for these patients using FHIR APIs with OAuth2.0 authentication and passes the feature set to COMPOSER. Hourly frequency was selected to ensure adequate data availability for a prediction. The resulting sepsis risk score and the top features driving the recommendation are then written to a flowsheet within the EHR using an HL7v2 outbound message. This flowsheet triggers a nurse-facing BPA (Fig. 4) on the chart open which alerts the caregiver that the patient is at risk of developing severe sepsis and provides the model’s top reasons. The nurse could acknowledge the alert by selecting one of the four options: (i) no infection suspected, (ii) sepsis treatment/workup in progress, or (iii) will notify MD immediately. If a nurse exited the patient’s chart without selecting an option, we recorded this as “no acknowledgement”. If the “will notify MD immediately” option is selected, the nurse can use a ‘secure chat’ feature to contact the provider from within the BPA to discuss the care of the patient.

Prospectively deployed algorithms are susceptible to model drift in which their performance degrades overtime due to changes in the patient population or treatment practices^41^. To detect this possibility, we implemented a data quality dashboard that tracks the median values of all input features to ensure they are within their upper and lower process control limits (based on the upper and lower quantiles from the training cohort). We further evaluate model performance, such as sensitivity and positive predictive value (PPV), biweekly to ensure there is no degradation in COMPOSER performance (Supplementary Note 3). We established a Predetermined Change Control Plan (PCCP) to trigger model retraining if the performance drops below predetermined thresholds, although this has not been required as of the time of this reporting.

### Implementation of COMPOSER into our electronic health record

Implementation was completed in various stages according to the EPIS (exploration, preparation, implementation, sustainment) framework, with frequent feedback provided to nursing staff during the implementation and sustainment periods^42^. Early stages in the Exploration stage began 2–3 years prior to model deployment with significant institutional support at the departmental and health system level. Preparation began approximately 6 months prior to the go-live date. Included in this was the creation of a multidisciplinary team to guide implementation, surveys of the nursing staff to identify specific needs, educational sessions, and iterative changes to the BPA from end-users. We employed a “silent mode trial” in which COMPOSER outputs were reviewed in real-time by a team of physicians to assess the accuracy and usefulness of the alerts. Adjustments to the algorithm were iteratively made based on these reviews to improve the timeliness and appropriateness of the alerts. During the Implementation phase, frequent feedback and education were provided to nursing staff on the COMPOSER model. In final form, our BPA would fire for all adult (at least 18 years old) patients who were receiving care in the ED with a score above the threshold and the following exclusions: patient discharged or deceased, comfort care measures initiated, patient no longer under the care of ED nurses, or a sepsis bundle had previously been instituted during their stay. Nurses would have to have the patient’s chart open for it to fire. The lockout periods for each acknowledgement reason were: “No infection suspected” 8 h; “Will notify MD immediately” 12 h; Sepsis treatment/work-up in progress” 12 h. We defined our pre-intervention time period from January 1st, 2021 to December 6th, 2022. COMPOSER went live December 7th, 2022. Our post-implementation phase was from December 7th, 2022 until April 30th, 2023.

### Primary and secondary outcomes

Our primary outcome was in-hospital mortality. Secondary outcomes included: compliance with our sepsis bundle (initial and repeat serum lactate if initial lactate >2 mmol/L, initiation and completion of a 30 mL/kg crystalloid fluid bolus, checking of blood cultures prior to antibiotics, and initiation of intravenous antibiotics within 3 h of time of sepsis), 72-h change in sequential organ failure assessment (SOFA) score following sepsis onset, ICU admission, and ICU-free days. ICU-free days were calculated as 30 less the number of ICU days with in-hospital death and stays longer than 30 days were fixed at 0. For patients who died in the ICU, this value is 0. For example, a patient who is in the ICU for 4 days and survives would have a value of 26. Patients who either die in the ICU or in the ICU for > 29 days have a value of 0.

### Statistical methods

Descriptive statistics were provided as indicated. Differences between the pre-intervention and post-intervention cohort were assessed with Kruskal–Wallis rank sum tests on continuous variables and Pearson’s chi-squared tests on categorical variables and significance was assessed at a *P*-value of 0.05. All statistical analyses were performed using the R statistical software version 4.0.4 and the CausalImpact package version 1.2.7^43,44^.

To estimate the causal effect of the COMPOSER BPA intervention, we performed causal inference using a Bayesian structural time-series model^44^. This approach, pioneered by Brodersen et al. from Google Inc., has been widely used to assess the impact of advertisement campaigns on product sales and the effect of economic changes on markets^45–47^. Here, we apply it to patient outcomes data to assess the impact of the COMPOSER algorithm adjusted for confounders. Briefly, a state-space model is trained on the control time-series prior to the intervention of interest. The observed outcome is modeled as a function of the latent state and Gaussian noise, with the latent state modeled by a local linear trend, in addition to a linear regression on the model covariates. In this work, we assume the effect of the regression coefficients on the outcome of interest is independent of time and the static regression parameters are sampled from a spike-and-slab prior distribution. Posterior inference is then performed on the post-intervention time-series to estimate the counterfactual outcome if the intervention had not been introduced. Under the assumption that the response variable in the control time-series is independent of the intervention, the difference between the model prediction and the observed value is a probability density of the causal impact of the intervention over time (See Supplementary Note 3 for more details). Additionally, model residuals were evaluated using quantile-quantile and autocorrelation plots to ensure adequate modeling of the time-series information.

Emergency department volume, sex, baseline SOFA, comorbidity burden via the Elixhauser comorbidity score, age, COVID-19 infection status, ED location (La Jolla or Hillcrest), local trends, and season were included as covariates in the Bayesian structural time-series model and 1000 samples of Markov Chain Monte Carlo were used for posterior inference. No imputation was performed since all covariates were fully observed. There were no missing values present in the covariates. We included seasons and local trends (e.g., ED volume) as prior data suggest outcomes of sepsis patients are worse in the winter and may be impacted by high patient volumes^48,49^. Outcomes were predicted at a monthly resolution to reduce the influence of random fluctuations on the outcome variable. We plotted the model predictions and true outcomes data, the pointwise difference between the two, and the cumulative difference across the post-intervention period and assessed significance against the 95% confidence intervals.

We further evaluated the association of alert acknowledgement and sepsis intervention, measured by the time from ED triage to the administration of antibiotics. We adjusted for the aforementioned confounders and performed a two-sided t-test on time-to-antibiotics as a function of acknowledgement reason.

### Reporting summary

Further information on research design is available in the Nature Research Reporting Summary linked to this article.

### Supplementary information

Supplemental Material

Reporting Summary

## Data Availability

Access to the de-identified UCSD cohort can be made available by contacting the corresponding author and via approval from the UCSD Institutional Review Boards (IRB) and Health Data Oversight Committee (HDOC).

## Code availability

Access to the R code used in this research is available upon request to the corresponding author. Details on the Causal Impact implementation can be found here: https://google.github.io/CausalImpact/CausalImpact.html.
