# Author Correction: Impact of a deep learning sepsis prediction model on quality of care and survival

**DOI:** 10.1038/s41746-024-01149-x

**Published:** 2024-06-12

**Authors:** Aaron Boussina, Supreeth P. Shashikumar, Atul Malhotra, Robert L. Owens, Robert El-Kareh, Christopher A. Longhurst, Kimberly Quintero, Allison Donahue, Theodore C. Chan, Shamim Nemati, Gabriel Wardi

**Affiliations:** 1 Department of Medicine, University of California San Diego, San Diego, CA USA; 2 Department of Quality, University of California San Diego, San Diego, CA USA; 3 Department of Emergency Medicine, University of California San Diego, San Diego, CA USA

**Keywords:** Prognosis, Preventive medicine

Correction to: *npj Digital Medicine* 10.1038/s41746-023-00986-6, published online 23 January 2024

Table 3 and the citation in the text were missing in this article. The original article has been corrected.

