# Supplementary material for: Impact of a deep learning sepsis prediction model on quality of care and survival
Source: NPJ Digit Med. 2024 Jan 23;7:14. doi: 10.1038/s41746-023-00986-6 (PMC10805720; doi:10.1038/s41746-023-00986-6)
Supplement: Supplementary file 1 — Supplemental Material [file 41746_2023_986_MOESM1_ESM.docx]

# **Supplemental Contents**

## **Supplementary Note 1.**

## STROBE Checklist.

## STROBE Statement—checklist of items that should be included in reports of observational studies

##

|  | Item No | Recommendation | Page No |
| --- | --- | --- | --- |
| Title and abstract | 1 | (*a*) Indicate the study’s design with a commonly used term in the title or the abstract | 1 |
|  |  | (*b*) Provide in the abstract an informative and balanced summary of what was done and what was found | 2-3 |
| Introduction | | | |
| Background/rationale | 2 | Explain the scientific background and rationale for the investigation being reported | 3 |
| Objectives | 3 | State specific objectives, including any prespecified hypotheses | 3 |
| Methods | | | |
| Study design | 4 | Present key elements of study design early in the paper | 4 |
| Setting | 5 | Describe the setting, locations, and relevant dates, including periods of recruitment, exposure, follow-up, and data collection | 5 |
| Participants | 6 | (*a*) *Cohort study*—Give the eligibility criteria, and the sources and methods of selection of participants. Describe methods of follow-up*Case-control study*—Give the eligibility criteria, and the sources and methods of case ascertainment and control selection. Give the rationale for the choice of cases and controls*Cross-sectional study*—Give the eligibility criteria, and the sources and methods of selection of participants | 6 |
|  |  | (*b*) *Cohort study*—For matched studies, give matching criteria and number of exposed and unexposed*Case-control study*—For matched studies, give matching criteria and the number of controls per case | 6 |
| Variables | 7 | Clearly define all outcomes, exposures, predictors, potential confounders, and effect modifiers. Give diagnostic criteria, if applicable | 7 |
| Data sources/ measurement | 8* | For each variable of interest, give sources of data and details of methods of assessment (measurement). Describe comparability of assessment methods if there is more than one group | *7-8* |
| Bias | 9 | Describe any efforts to address potential sources of bias | 7-8 |
| Study size | 10 | Explain how the study size was arrived at | N/A |
| Quantitative variables | 11 | Explain how quantitative variables were handled in the analyses. If applicable, describe which groupings were chosen and why | 8 |
| Statistical methods | 12 | (*a*) Describe all statistical methods, including those used to control for confounding | 7-8 |
|  |  | (*b*) Describe any methods used to examine subgroups and interactions | 8 |
|  |  | (*c*) Explain how missing data were addressed | 8 |
|  |  | (*d*) *Cohort study*—If applicable, explain how loss to follow-up was addressed*Case-control study*—If applicable, explain how matching of cases and controls was addressed*Cross-sectional study*—If applicable, describe analytical methods taking account of sampling strategy | N/A |
|  |  | (*e*) Describe any sensitivity analyses | 8 |

## Continued on next page

| Results | | | |
| --- | --- | --- | --- |
| Participants | 13* | (a) Report numbers of individuals at each stage of study—eg numbers potentially eligible, examined for eligibility, confirmed eligible, included in the study, completing follow-up, and analysed | 9 |
|  |  | (b) Give reasons for non-participation at each stage | 9 |
|  |  | (c) Consider use of a flow diagram | N/A |
| Descriptive data | 14* | (a) Give characteristics of study participants (eg demographic, clinical, social) and information on exposures and potential confounders | 9-10 |
|  |  | (b) Indicate number of participants with missing data for each variable of interest | 8 |
|  |  | (c) *Cohort study*—Summarise follow-up time (eg, average and total amount) | N/A |
| Outcome data | 15* | *Cohort study*—Report numbers of outcome events or summary measures over time | *11-12* |
|  |  | *Case-control study—*Report numbers in each exposure category, or summary measures of exposure | *N/A* |
|  |  | *Cross-sectional study—*Report numbers of outcome events or summary measures | *N/A* |
| Main results | 16 | (*a*) Give unadjusted estimates and, if applicable, confounder-adjusted estimates and their precision (eg, 95% confidence interval). Make clear which confounders were adjusted for and why they were included | 11-16 |
|  |  | (*b*) Report category boundaries when continuous variables were categorized | 11-16 |
|  |  | (*c*) If relevant, consider translating estimates of relative risk into absolute risk for a meaningful time period | 11-12 |
| Other analyses | 17 | Report other analyses done—eg analyses of subgroups and interactions, and sensitivity analyses | 12 |
| Discussion | | | |
| Key results | 18 | Summarise key results with reference to study objectives | 17 |
| Limitations | 19 | Discuss limitations of the study, taking into account sources of potential bias or imprecision. Discuss both direction and magnitude of any potential bias | 19 |
| Interpretation | 20 | Give a cautious overall interpretation of results considering objectives, limitations, multiplicity of analyses, results from similar studies, and other relevant evidence | 17-19 |
| Generalisability | 21 | Discuss the generalisability (external validity) of the study results | 19-20 |
| Other information | | | |
| Funding | 22 | Give the source of funding and the role of the funders for the present study and, if applicable, for the original study on which the present article is based | 20 |

##

## *Give information separately for cases and controls in case-control studies and, if applicable, for exposed and unexposed groups in cohort and cross-sectional studies.

##

## Note: An Explanation and Elaboration article discusses each checklist item and gives methodological background and published examples of transparent reporting. The STROBE checklist is best used in conjunction with this article (freely available on the Web sites of PLoS Medicine at http://www.plosmedicine.org/, Annals of Internal Medicine at http://www.annals.org/, and Epidemiology at http://www.epidem.com/). Information on the STROBE Initiative is available at www.strobe-statement.org.

## **Supplementary Note 2. Cloud-based infrastructure for model deployment.**


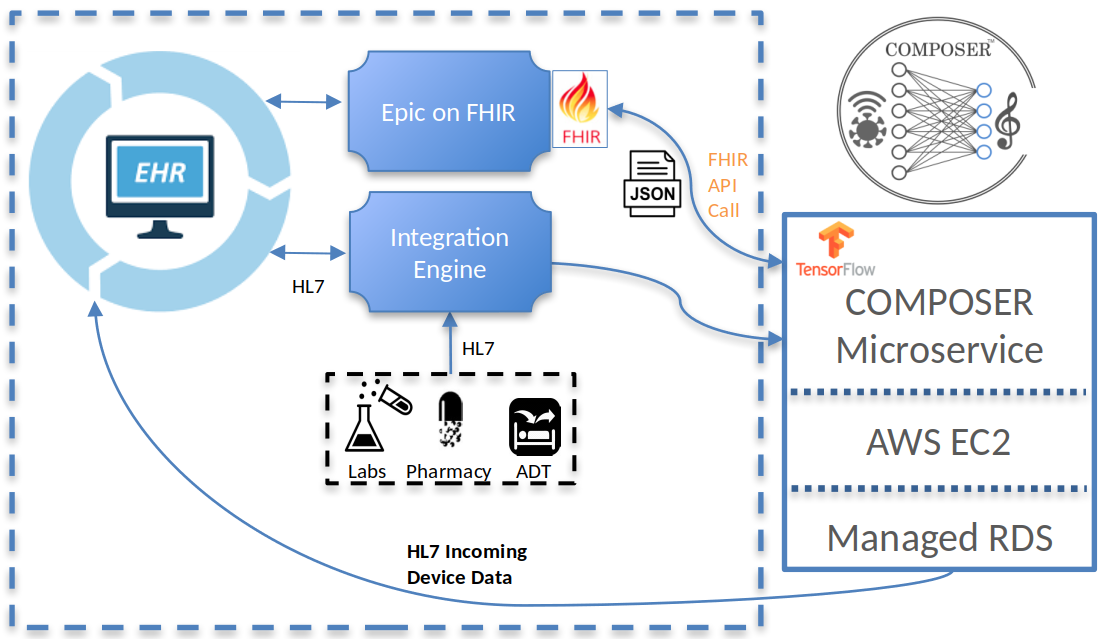


**Supplementary Figure 1. System Diagram of the Deep Learning Platform.** The core application runs on an elastic computing virtual machine with an auto-scaling group to ensure fault tolerance and high availability. HL7 message feeds and FHIR JSON responses are stored in managed relational databases. Model predictions are sent back to the EHR through an HL7 device data interface. HL7 = health level 7; AWS = Amazon Web Services; FHIR = Fast Healthcare Interoperability Resources; ADT = admission discharge transfer; RDS = relational database service; EC2 = Elastic Compute Cloud

## **Supplementary Note 3. Algorithm Performance Monitoring**

The cloud application described in Supplementary Note 2 captures the HL7v2 and FHIR feeds for a patient through discharge.  This enables identification of patients meeting Sepsis-3 criteria as described in the main text.  The COMPOSER performance monitoring system tags these patients for Sepsis-3 on a biweekly schedule and compares these labels against the COMPOSER BPA firings, allowing for automated monitoring of the algorithm sensitivity, specificity, and PPV.  We visualize these metrics, as well as the input feature distributions, in a data quality dashboard (Supplementary Figure 2) and generate an AWS CloudWatch alert for algorithm retraining when the PPV drops below a threshold of 20%.  Additionally, the system generates PagerDuty® alerts whenever the FHIR connection is interrupted, an outbound HL7v2 message fails to be received, or the inbound ADT connection is severed.


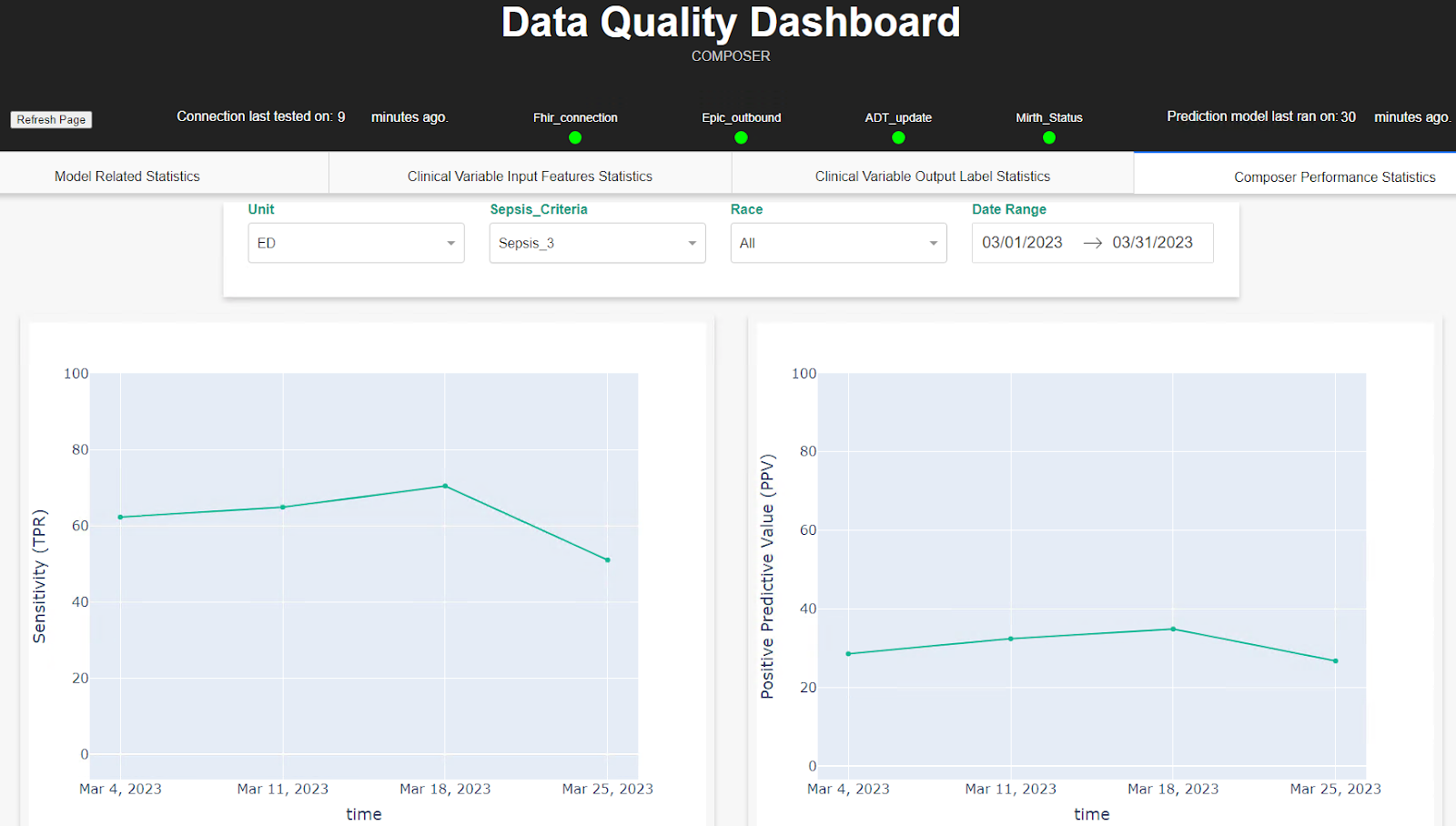


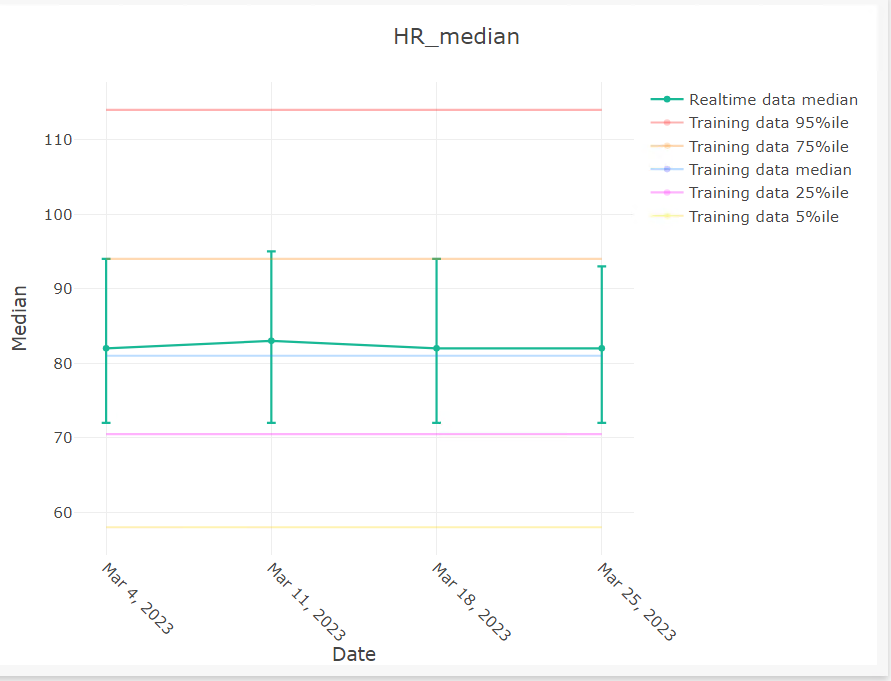

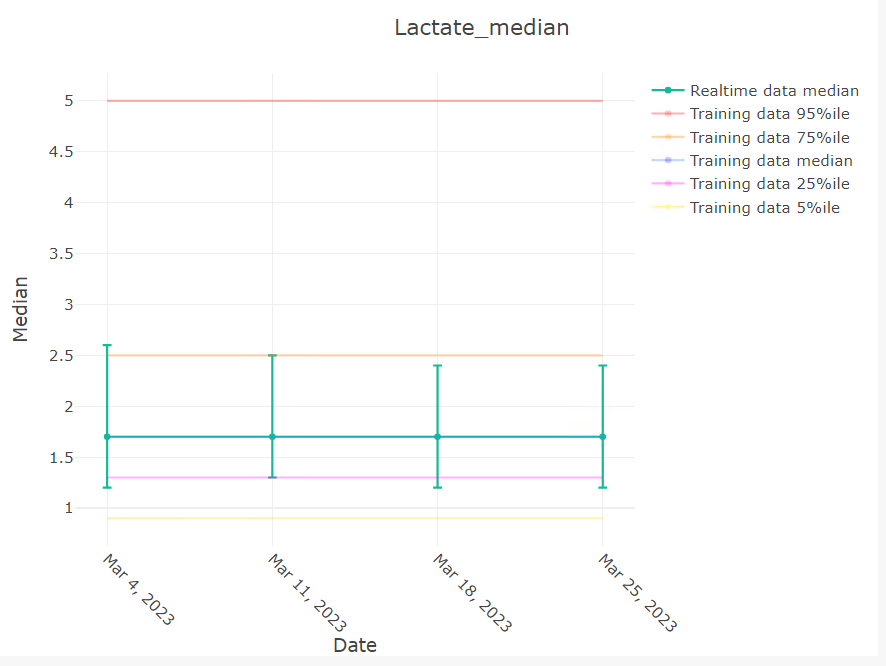


**Supplementary Figure 2.** Examples of monitoring of Input features and performance outputs from the COMPOSER data quality dashboard.

## **Supplementary Note 4. Causal impact analysis with Bayesian structural time-series models.**

The Bayesian structural time-series model used in this work is described by Supplementary Equations 1 - 7 and Supplementary Fig 2. The observed outcome y_t_ is modeled as the combination of a local state (𝜇_t_), covariate regression (x_t_^T^𝛽), seasonality ($\gamma$_t_), and Gaussian noise (𝜀_t_). The state components (𝜇_t_, 𝛿_t_) are modeled by a local linear trend which evolve according to Gaussian random walks. A spike-and-slab prior is used on the regression parameters (𝛽) which are integrated out, enabling the uncertainty of covariate influence to be accounted for in the model inference. Additionally, a hyperparameter search is performed on the order of seasonality parameter (S) to ensure whiteness of the autocorrelation of the residuals (i.e., any remaining unexplained residuals are uncorrelated gaussian noise). Posterior inference is carried out by drawing 1000 samples using Markov Chain Monte Carlo, where model parameters are sampled from their joint posterior distribution given the data. For each set of sampled parameters, a prediction y_i_​ is generated. The distribution of these predictions across all 1000 samples provides an estimation of the prediction uncertainty.

|  | 1. y_t_ = x_t_^T^𝛽 + 𝜇_t_ + $\gamma$_t_ + 𝜀_t_ 2. 𝜇_t_ = 𝜇_t-1_ + 𝛿_t-1_ + 𝜂_𝜇,t_ 3. 𝛿_t_ = 𝛿_t-1_ + 𝜂_𝛿,t_ 4. 𝜀_t_ ~ N(0, 𝜎_t_^2^) 5. $\gamma$_t+1_ = $-\sum_{s=0}^{S-2} {\gamma_{t-s}+\eta_{\gamma,t}}{}$ 6. 𝜂_𝛿,t_ ~ N(0, 𝜎_𝛿_^2^) 7. 𝜂_𝜇,t_ ~ N(0, 𝜎_𝜇_^2^) |
| --- | --- |

**
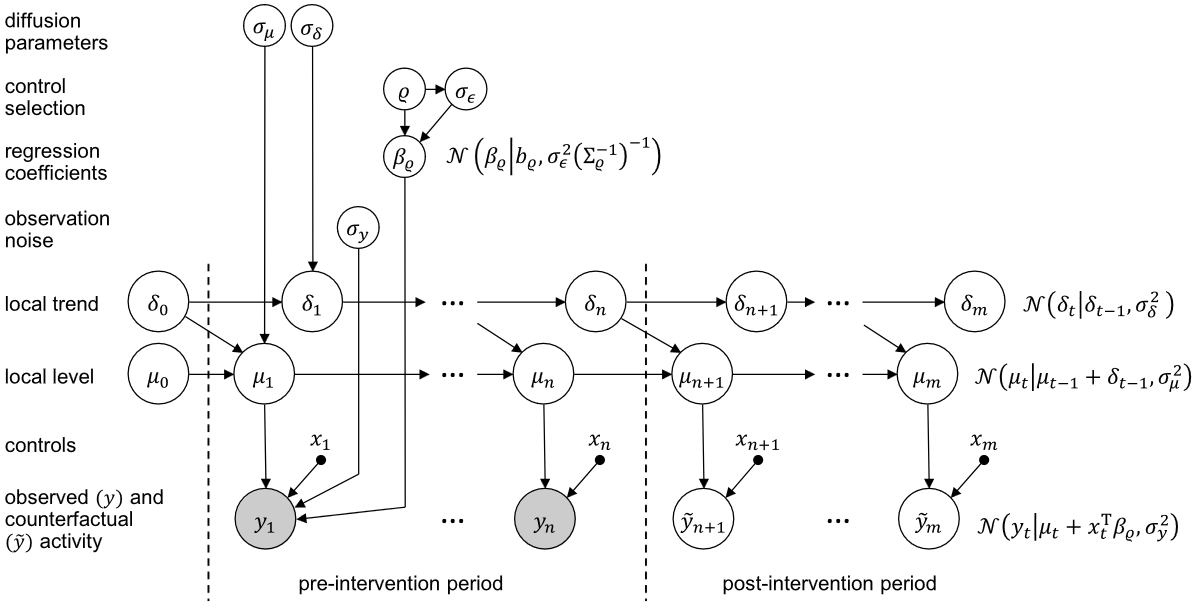
**

**Supplementary Figure 3.** Graphical model of the state-space model, adapted from Brodersen, et al.^22^

##

## **Supplementary Note 5. Comparison between the two emergency departments.**

**Supplementary Table 1. Demographics and baseline characteristics of septic patients at the La Jolla (LJ) and Hillcrest (HC) emergency departments.**

|  | **Total** | **LJ Emergency Department** | **HC Emergency Department** | ***P***  **Value^a^** |
| --- | --- | --- | --- | --- |
| **Characteristic** | | | | |
| Number of Patients, N (%) | 6217 (100%) | 3002 (48.3%) | 3215 (51.7%) | - |
| Age, Mean (SD) | 63 (17.1) | 65 (17.1) | 61 (16.8) | < .001 |
| Sex, N (%) | | | | |
| Male | 3592 (57.8%) | 1586 (52.8%) | 2006 (62.4%) | - |
| Female | 2625 (42.2%) | 1416 (47.2%) | 1209 (37.6%) | - |
| Ethnicity |  |  |  |  |
| Asian | 530 (8.5%) | 365 (12.2%) | 165 (5.1%) | - |
| Black or African American | 639 (10.3%) | 141 (4.7%) | 498 (15.5%) | - |
| White | 2983 (48%) | 1564 (52.1%) | 1419 (44.1%) | - |
| Other^c^ | 2065 (33.2%) | 932 (31%) | 1133 (35.2%) | - |
| Ethnic Group |  |  |  | - |
| Hispanic/Latino | 1756 (28.2%) | 805 (26.8%) | 951 (29.6%) | - |
| Not Hispanic/Latino | 4461 (71.8%) | 2197 (73.2%) | 2264 (70.4%) | - |
| Cancer Patients, N (%) | 1602 (26.6%) | 1211 (40.3%) | 391 (12.1%) | < .001 |
| **Organ Dysfunction** | | | | |
| Elixhauser Comorbidity Index,  Median (IQR) | 5 (0-13) | 7 (0-16) | 0 (0-11) | < .001 |
| SOFA Score at Time of Sepsis,  Median (IQR) | 0.7 (1.3) | 0.5 (0.98) | 0.9 (1.52) | < .001 |
| **Lab Values** |  |  |  |  |
| Lactate at Time of Sepsis | 2.4 (1.6 - 4.3) | 2.15 (1.5 - 3.5) | 2.7 (1.7 - 5.0) | < .001 |
| **Interventions** | | | | |
| Mechanical Ventilation, N (%)^d^ | 1,035 (16.6%) | 849 (16.8%) | 186 (16.1%) | < .001 |
| Administration of  Vasoactive Medications, N (%)^d^ | 424 (6.8%) | 345 (6.8%) | 79 (6.9%) | 0.008 |

## **^a^**P values for continuous variables are based on Kruskal-Wallis rank sum tests. P values for categorical variables are based on Pearson’s chi-squared tests.

^b^Ethnicity is self-reported.

**^c^**Other ethnicity corresponds to respondant selecting Native Hawaiian or Other Pacific Islander, American Indian or Alaska Native, Other Race or Mixed Race, or Unknown.

**^d^**Within 72-hours of ED arrival.

## **Supplementary Note 6. Diagnostic plots**


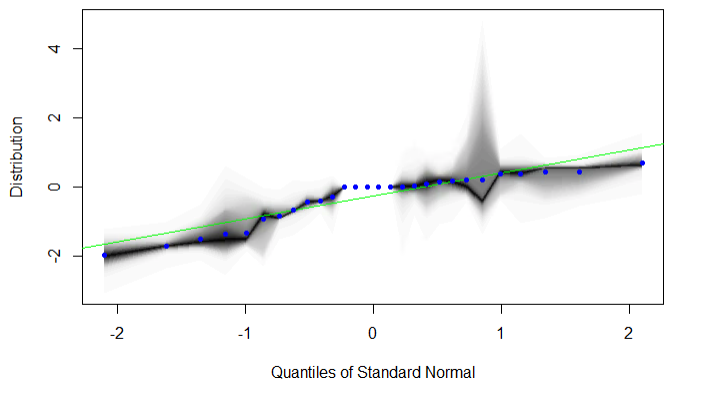


**Supplementary Figure 4.** **Residual quantile-quantile (QQ) plot for the mortality Bayesian structural time-series model.** Residuals from a well-fitted model are expected to be random Gaussian noise. The observed residuals for the sepsis mortality time-series model (Figure 2A) are well approximated by a normal distribution.

**
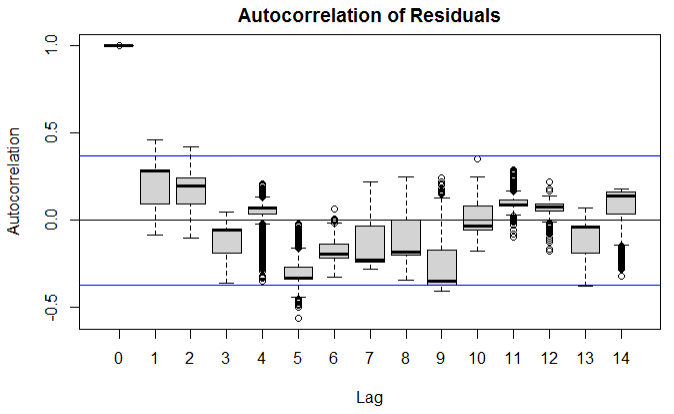
**

**Supplementary Figure 5. Boxplot of autocorrelation of residuals for the mortality Bayesian structural time-series model.** The center line of each box represents the median autocorrelation across 1000 runs of the Markov chain Monte Carlo algorithm. The box limits represent the upper and lower quantiles and the whiskers extend up to 1.5x the IQR with outliers displayed as individual points. There is no significant correlation between the residuals suggesting adequate fit of the time-series model.

##

##

##

##

## **Supplementary Note 7. Causal impact analysis of patient mortality by emergency department.
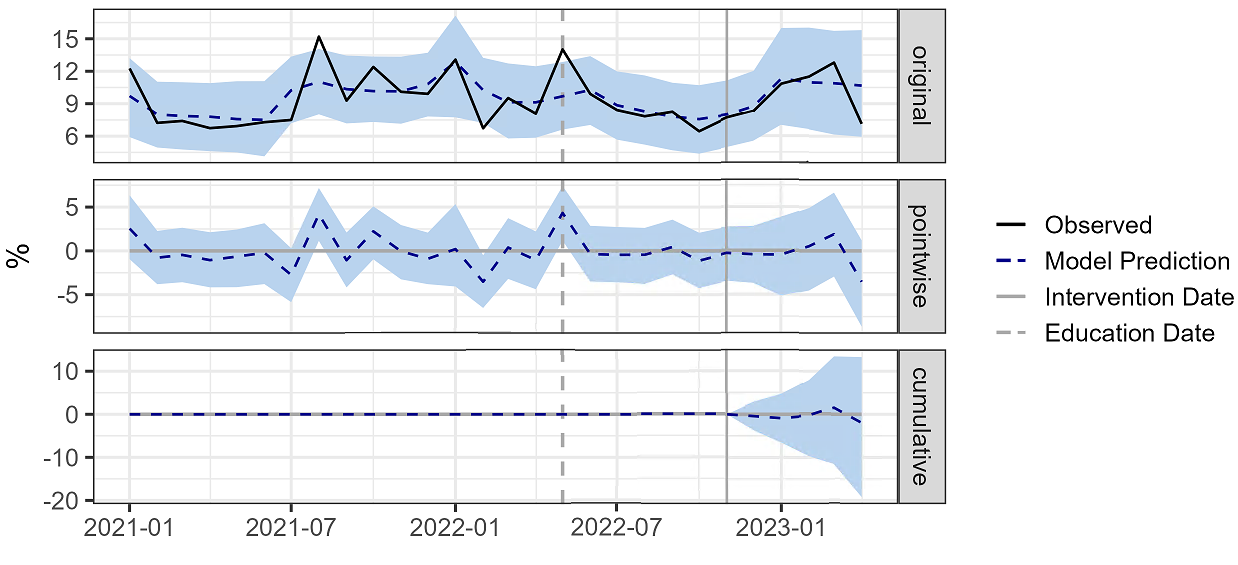
**

## **Supplementary Figure 6. Causal impact analysis of COMPOSER Best Practice Advisory on sepsis mortality at the La Jolla Emergency Department.** Plots of the causal impact analysis using a Bayesian structural time-series model. The top subpanel (“original”) shows the actual outcome (black) and the average model predictions (dashed blue) and 95% confidence limits (shaded blue) during the pre-intervention and post-intervention periods, indicated by the solid gray vertical line. The middle subpanel (“pointwise”) shows the difference between the model predictions and the observed outcome. The bottom subpanel (“cumulative”) shows the sum of the pointwise differences during the post-intervention period.

**
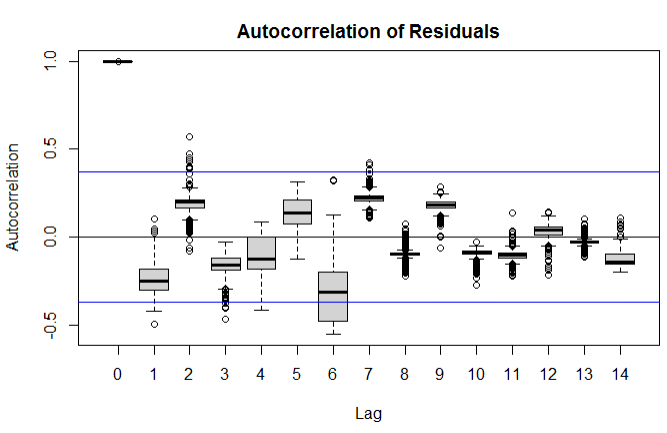
Supplementary Figure 7. Boxplot of autocorrelation of residuals for the mortality Bayesian structural time-series model for sepsis mortality at the La Jolla Emergency Department.** The center line of each box represents the median autocorrelation across 1000 runs of the Markov chain Monte Carlo algorithm. The box limits represent the upper and lower quantiles and the whiskers extend up to 1.5x the IQR with outliers displayed as individual points. There is no significant correlation between the residuals suggesting adequate fit of the time-series model.


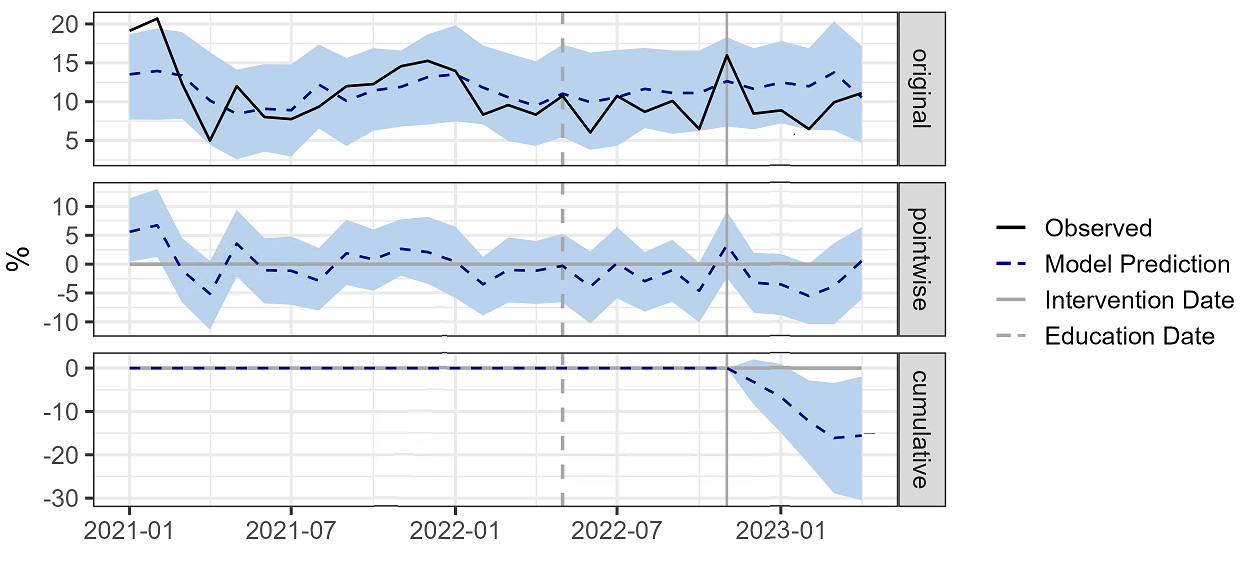
**Supplementary Figure 8. Causal impact analysis of COMPOSER Best Practice on sepsis mortality at the Hillcrest Emergency Department.** Plots of the causal impact analysis using a Bayesian structural time-series model. The top subpanel (“original”) shows the actual outcome (black) and the average model predictions (dashed blue) and 95% confidence limits (shaded blue) during the pre-intervention and post-intervention periods, indicated by the solid gray vertical line. The middle subpanel (“pointwise”) shows the difference between the model predictions and the observed outcome. The bottom subpanel (“cumulative”) shows the sum of the pointwise differences during the post-intervention period.

**
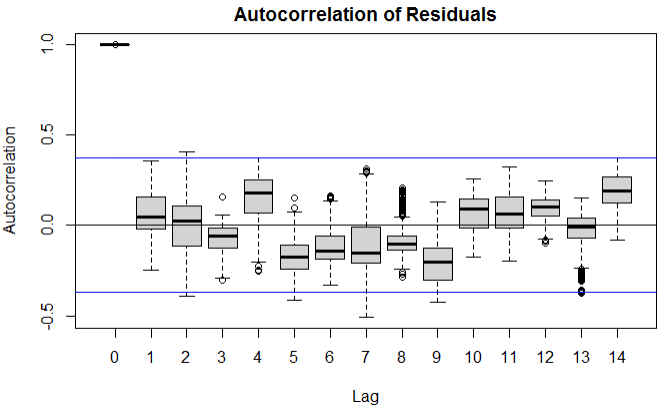
**

**Supplementary Figure 9. Boxplot of autocorrelation of residuals for the mortality Bayesian structural time-series model for sepsis mortality at the Hillcrest Emergency Department.** The center line of each box represents the median autocorrelation across 1000 runs of the Markov chain Monte Carlo algorithm. The box limits represent the upper and lower quantiles and the whiskers extend up to 1.5x the IQR with outliers displayed as individual points. There is no significant correlation between the residuals suggesting adequate fit of the time-series model.

## **Supplementary Note 8. Causal impact analysis of compliance with the individual sepsis bundle components.**


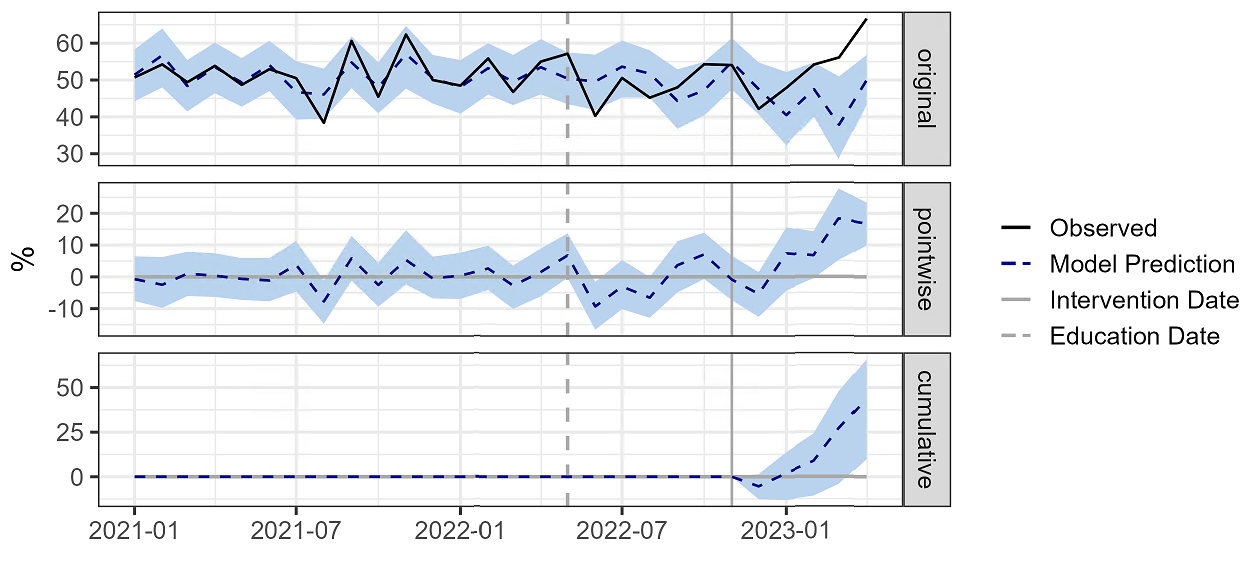


**Supplementary Figure 10. Causal impact analysis of COMPOSER Best Practice Advisory on sepsis bundle compliance rate at the La Jolla Emergency Department.** Plots of the causal impact analysis using a Bayesian structural time-series model. The top subpanel (“original”) shows the actual outcome (black) and the average model predictions (dashed blue) and 95% confidence limits (shaded blue) during the pre-intervention and post-intervention periods, indicated by the solid gray vertical line. The middle subpanel (“pointwise”) shows the difference between the model predictions and the observed outcome. The bottom subpanel (“cumulative”) shows the sum of the pointwise differences during the post-intervention period.

**
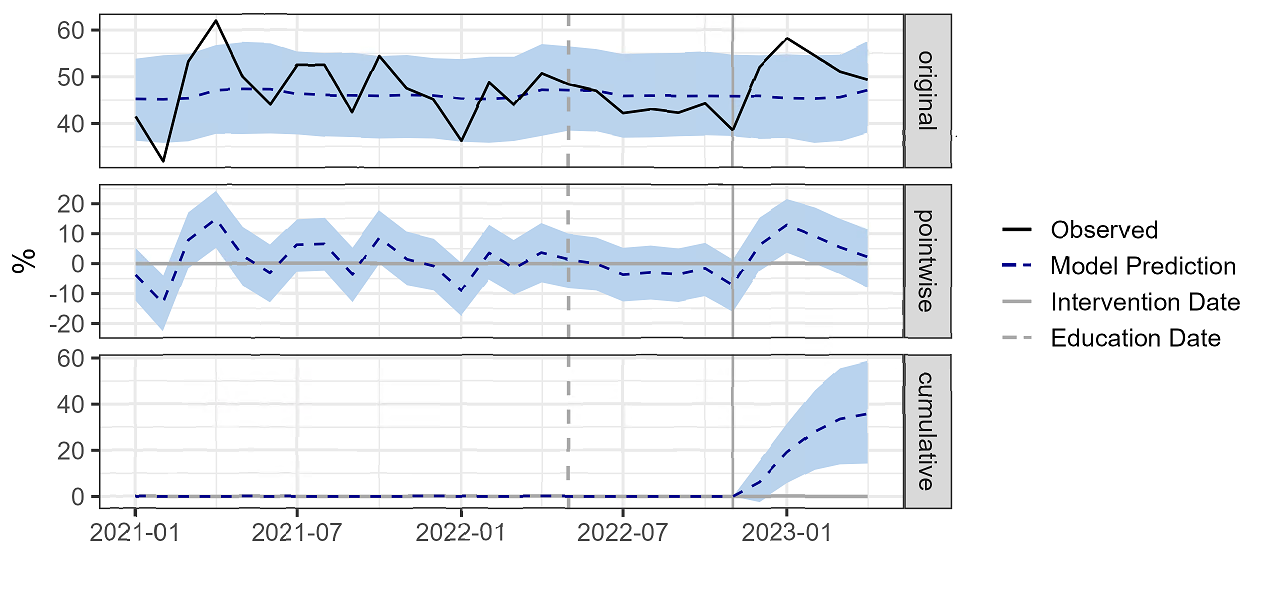
**

**Supplementary Figure 11. Causal impact analysis of COMPOSER Best Practice Advisory on sepsis bundle compliance rate at the Hillcrest Emergency Department.** Plots of the causal impact analysis using a Bayesian structural time-series model. The top subpanel (“original”) shows the actual outcome (black) and the average model predictions (dashed blue) and 95% confidence limits (shaded blue) during the pre-intervention and post-intervention periods, indicated by the solid gray vertical line. The middle subpanel (“pointwise”) shows the difference between the model predictions and the observed outcome. The bottom subpanel (“cumulative”) shows the sum of the pointwise differences during the post-intervention period.

**
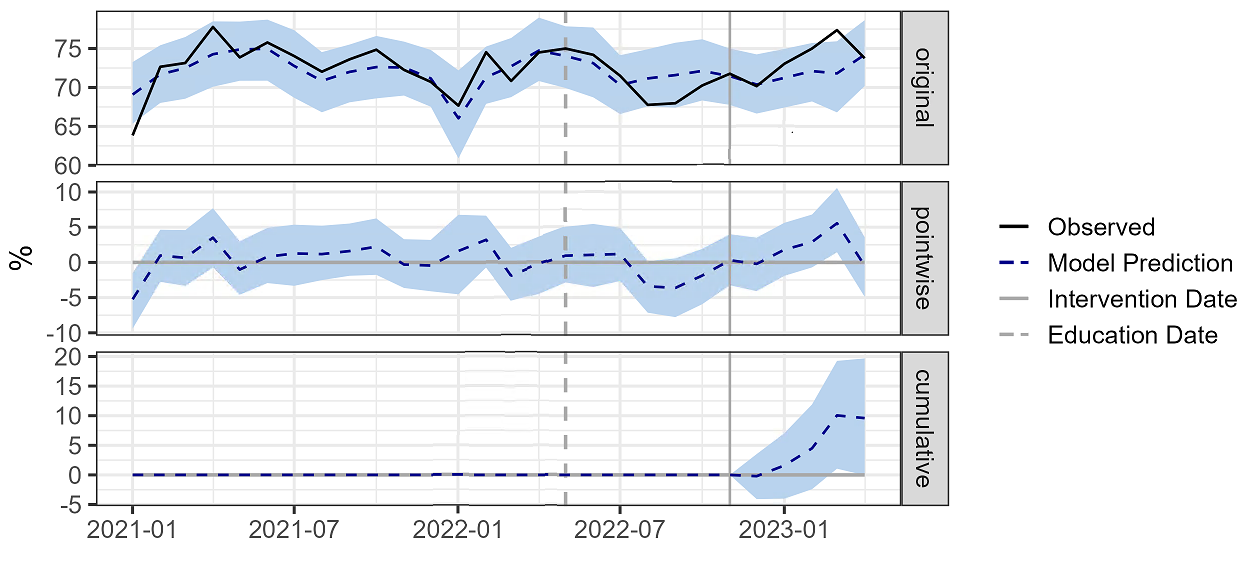
**

**Supplementary Figure 12. Blood cultures prior to antibiotics compliance rate.** Plots of the causal impact analysis using a Bayesian structural time-series model. The top subpanel (“original”) shows the actual outcome (black) and the average model predictions (dashed blue) and 95% confidence limits (shaded blue) during the pre-intervention and post-intervention periods, indicated by the solid gray vertical line. The middle subpanel (“pointwise”) shows the difference between the model predictions and the observed outcome. The bottom subpanel (“cumulative”) shows the sum of the pointwise differences during the post-intervention period.

**
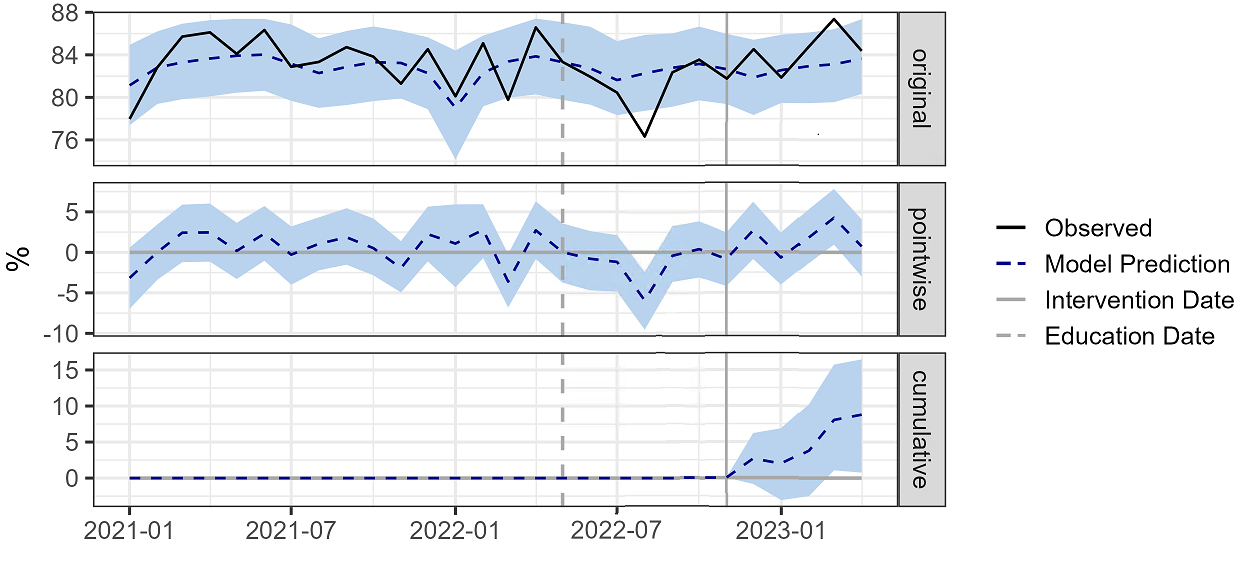
**

**Supplementary Figure 13. Rate of antibiotics administered within 24 hours prior and 3 hours after severe sepsis onset.** Plots of the causal impact analysis using a Bayesian structural time-series model. The top subpanel (“original”) shows the actual outcome (black) and the average model predictions (dashed blue) and 95% confidence limits (shaded blue) during the pre-intervention and post-intervention periods, indicated by the solid gray vertical line. The middle subpanel (“pointwise”) shows the difference between the model predictions and the observed outcome. The bottom subpanel (“cumulative”) shows the sum of the pointwise differences during the post-intervention period.

**
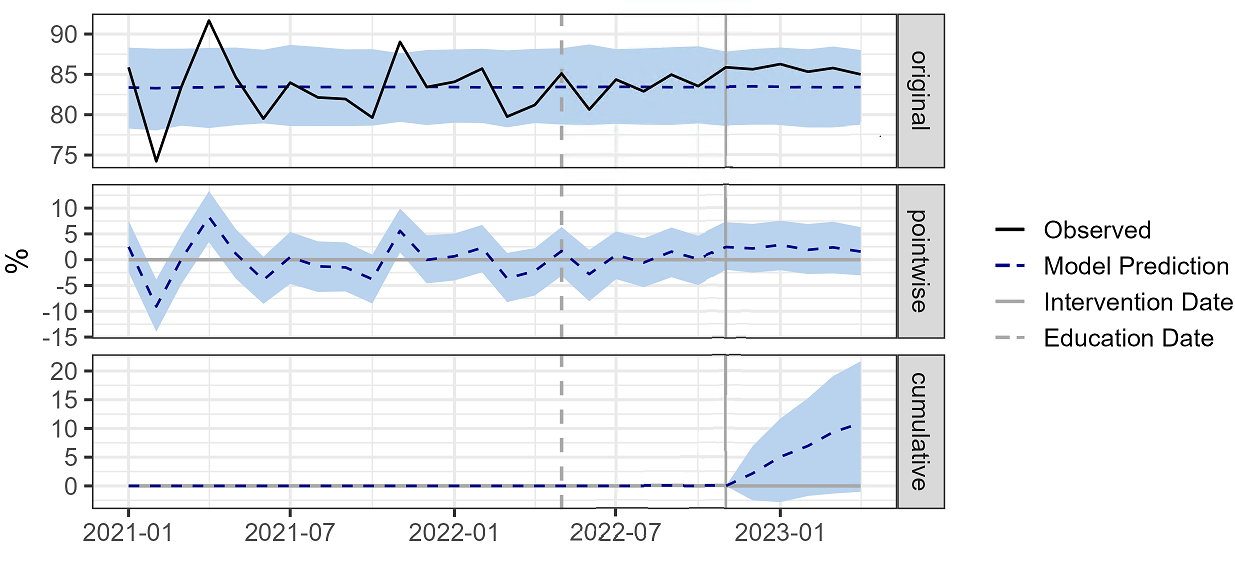
**

**Supplementary Figure 14. Rate of lactate measured within 6 hours prior and 3 hours after severe sepsis onset.** Plots of the causal impact analysis using a Bayesian structural time-series model. The top subpanel (“original”) shows the actual outcome (black) and the average model predictions (dashed blue) and 95% confidence limits (shaded blue) during the pre-intervention and post-intervention periods, indicated by the solid gray vertical line. The middle subpanel (“pointwise”) shows the difference between the model predictions and the observed outcome. The bottom subpanel (“cumulative”) shows the sum of the pointwise differences during the post-intervention period.

**
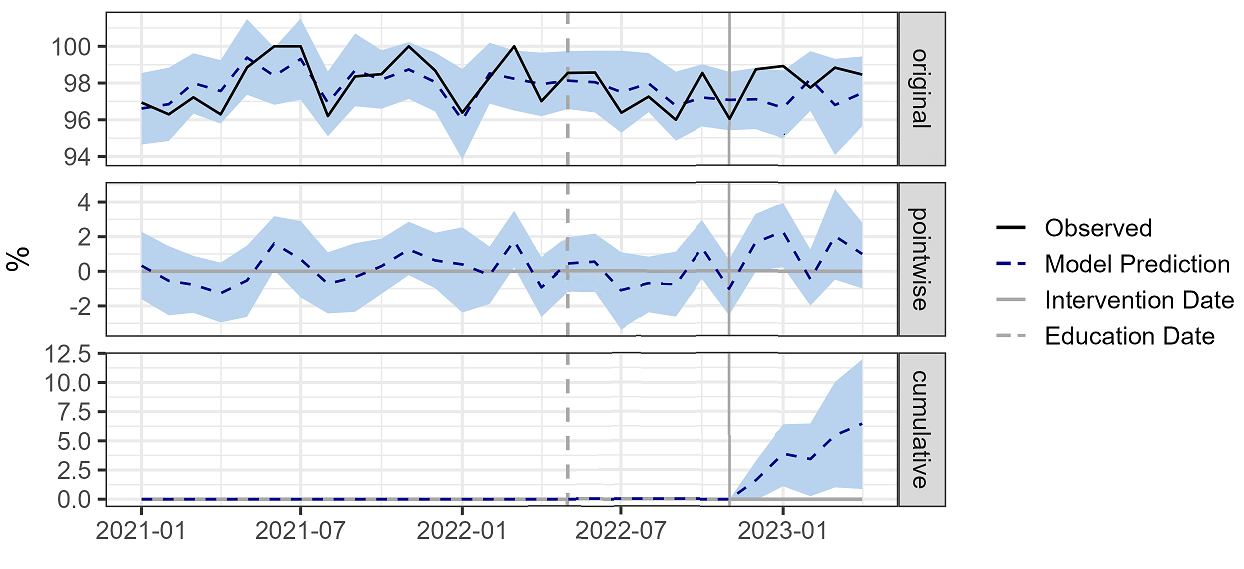
**

**Supplementary Figure 15. Rate of repeat lactate measured within 6 hours after severe sepsis onset if initial lactate is elevated.** Plots of the causal impact analysis using a Bayesian structural time-series model. The top subpanel (“original”) shows the actual outcome (black) and the average model predictions (dashed blue) and 95% confidence limits (shaded blue) during the pre-intervention and post-intervention periods, indicated by the solid gray vertical line. The middle subpanel (“pointwise”) shows the difference between the model predictions and the observed outcome. The bottom subpanel (“cumulative”) shows the sum of the pointwise differences during the post-intervention period.

**
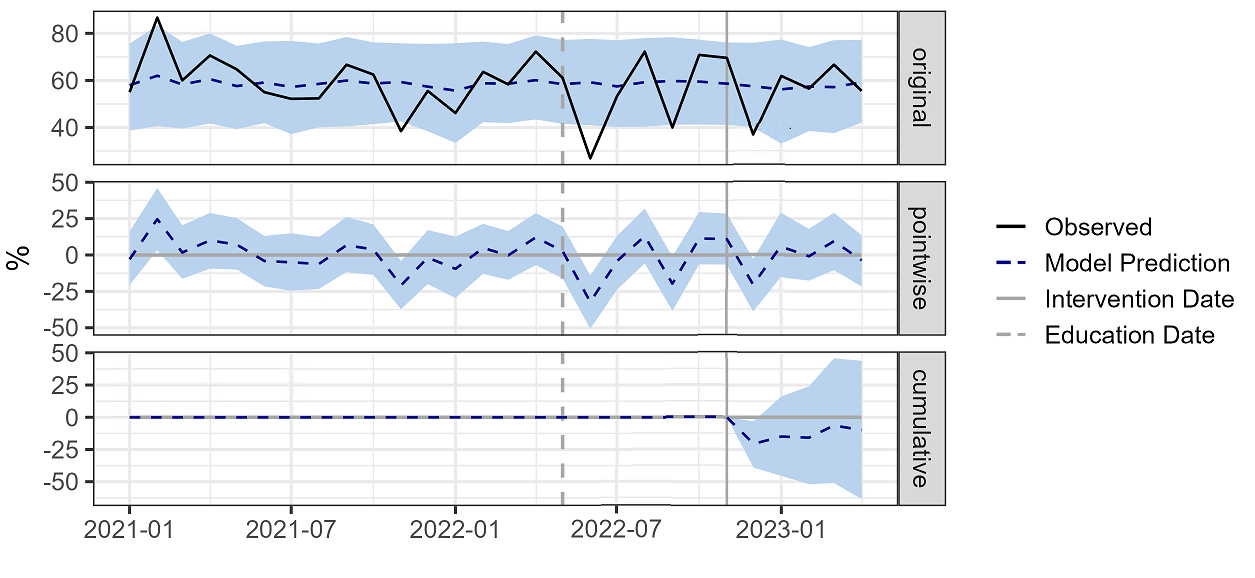
**

**Supplementary Figure 16. Rate of administration of vasoactive medications within 6 hours of septic shock.** Plots of the causal impact analysis using a Bayesian structural time-series model. The top subpanel (“original”) shows the actual outcome (black) and the average model predictions (dashed blue) and 95% confidence limits (shaded blue) during the pre-intervention and post-intervention periods, indicated by the solid gray vertical line. The middle subpanel (“pointwise”) shows the difference between the model predictions and the observed outcome. The bottom subpanel (“cumulative”) shows the sum of the pointwise differences during the post-intervention period.

**
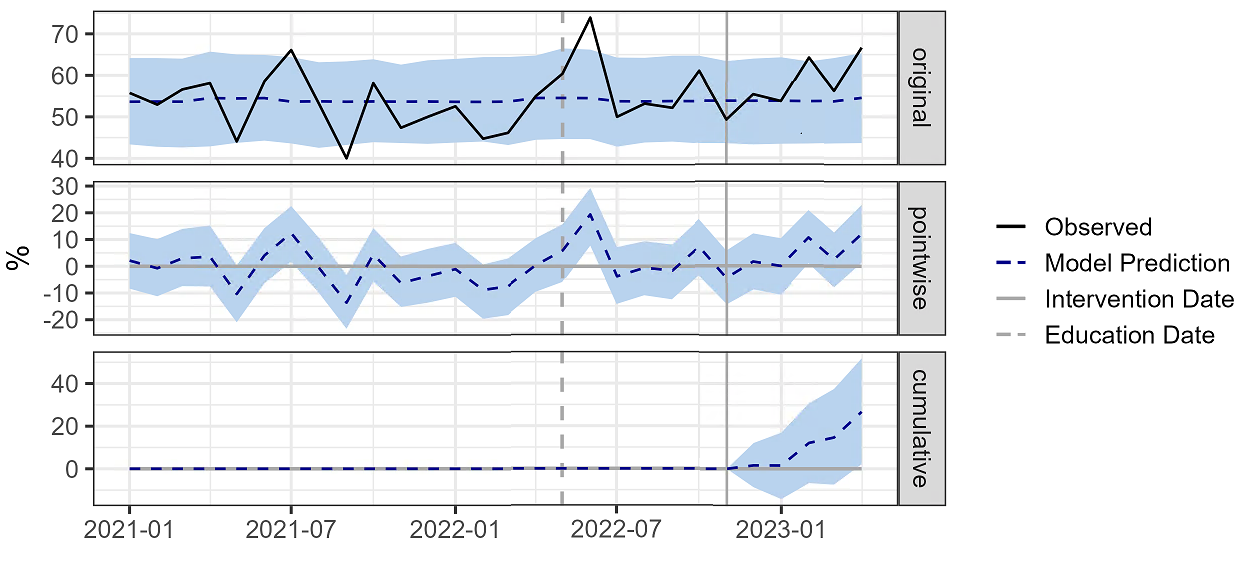
**

**Supplementary Figure 17. Rate of administration of 30cc/kg of fluids within 3 hours of presentation of septic shock or hypotension.** Plots of the causal impact analysis using a Bayesian structural time-series model. The top subpanel (“original”) shows the actual outcome (black) and the average model predictions (dashed blue) and 95% confidence limits (shaded blue) during the pre-intervention and post-intervention periods, indicated by the solid gray vertical line. The middle subpanel (“pointwise”) shows the difference between the model predictions and the observed outcome. The bottom subpanel (“cumulative”) shows the sum of the pointwise differences during the post-intervention period.

## **Supplementary Note 9. Causal impact analysis of change in sequential organ failure assessment (SOFA)score by emergency department.**

**
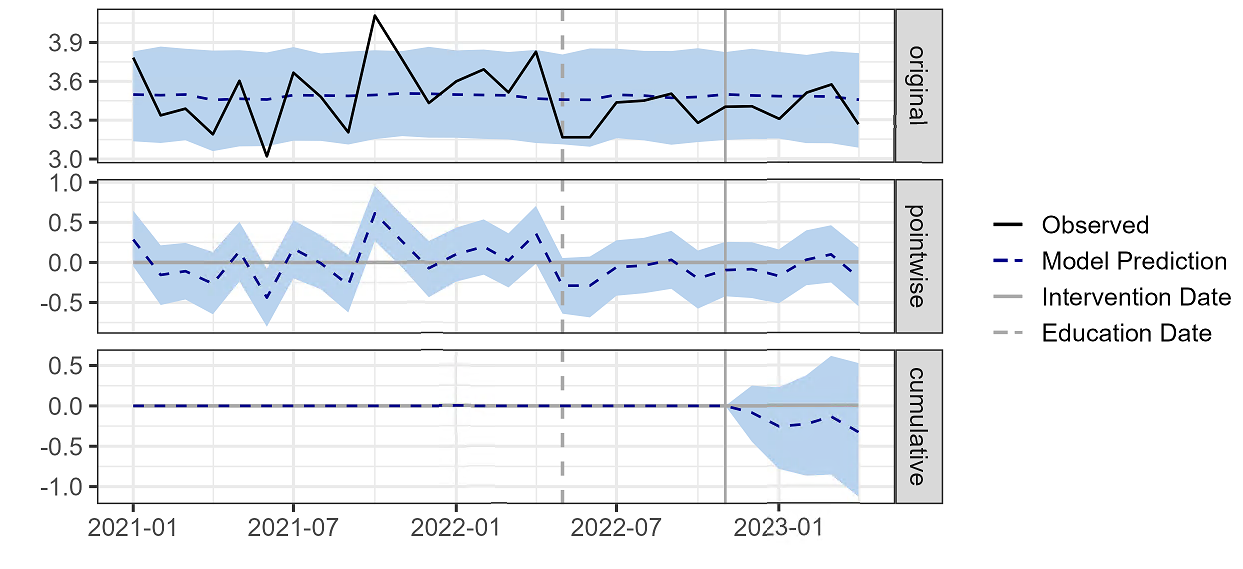
**

**Supplementary Figure 18. Causal impact analysis of change in sequential organ failure assessment (SOFA) score within 72 hours of sepsis onset at the La Jolla Emergency Department.** Plots of the causal impact analysis using a Bayesian structural time-series model. The top subpanel (“original”) shows the actual outcome (black) and the average model predictions (dashed blue) and 95% confidence limits (shaded blue) during the pre-intervention and post-intervention periods, indicated by the solid gray vertical line. The middle subpanel (“pointwise”) shows the difference between the model predictions and the observed outcome. The bottom subpanel (“cumulative”) shows the sum of the pointwise differences during the post-intervention period.

**
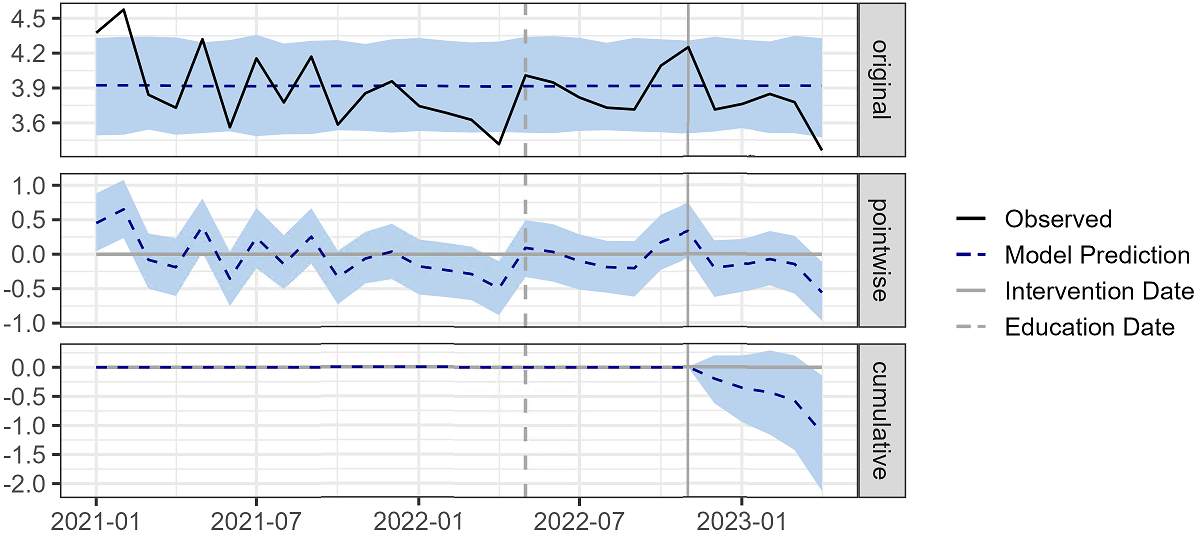
**

**Supplementary Figure 19. Causal impact analysis of change in sequential organ failure assessment (SOFA) score within 72 hours of sepsis onset at the Hillcrest Emergency Department.** Plots of the causal impact analysis using a Bayesian structural time-series model. The top subpanel (“original”) shows the actual outcome (black) and the average model predictions (dashed blue) and 95% confidence limits (shaded blue) during the pre-intervention and post-intervention periods, indicated by the solid gray vertical line. The middle subpanel (“pointwise”) shows the difference between the model predictions and the observed outcome. The bottom subpanel (“cumulative”) shows the sum of the pointwise differences during the post-intervention period.

## **Supplementary Note 10. Causal impact analysis of secondary outcomes.**

**
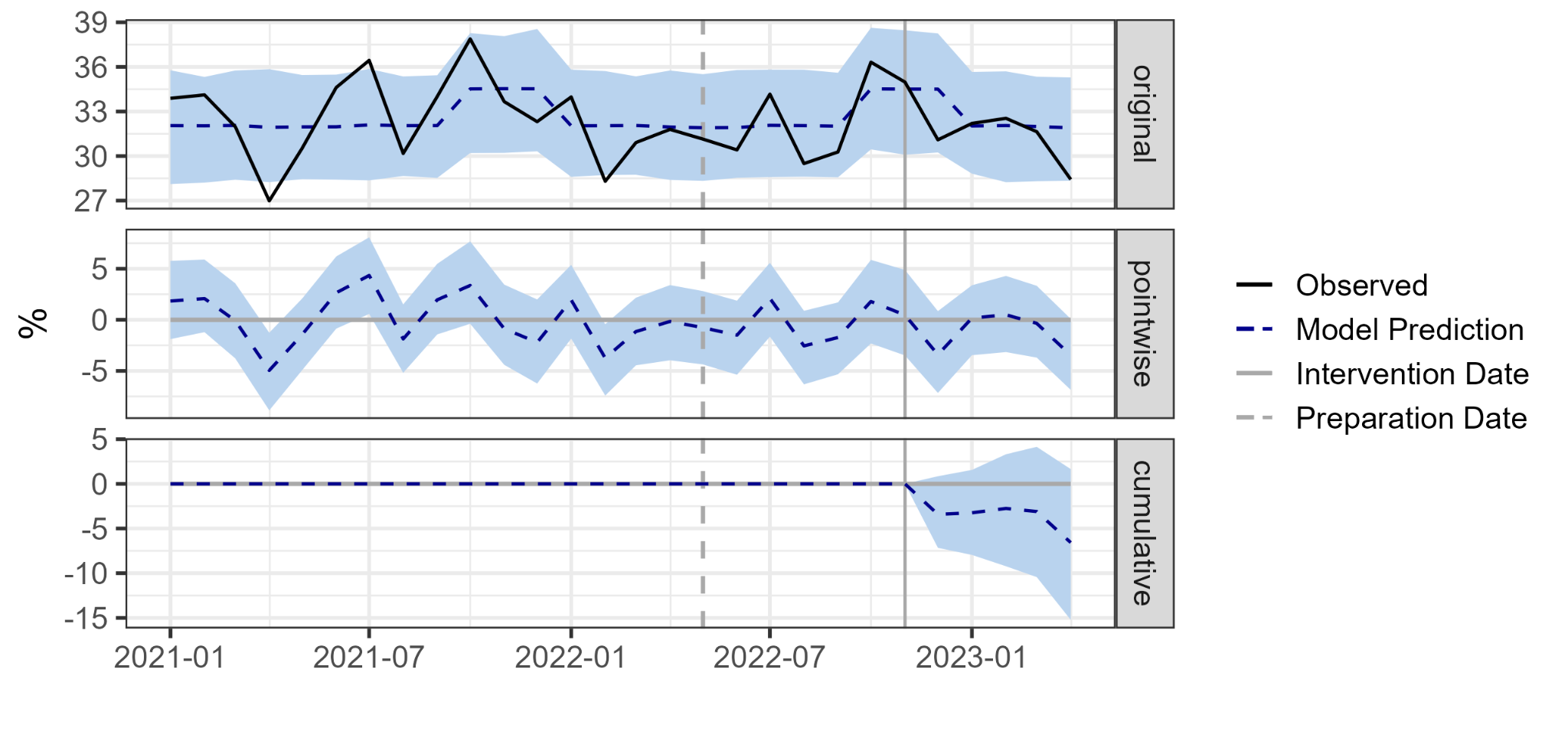
**

**Supplementary Figure 20. Causal impact analysis of sepsis intensive care unit (ICU) admissions from the emergency department.** Plots of the causal impact analysis using a Bayesian structural time-series model. The top subpanel (“original”) shows the actual outcome (black) and the average model predictions (dashed blue) and 95% confidence limits (shaded blue) during the pre-intervention and post-intervention periods, indicated by the solid gray vertical line. The middle subpanel (“pointwise”) shows the difference between the model predictions and the observed outcome. The bottom subpanel (“cumulative”) shows the sum of the pointwise differences during the post-intervention period.


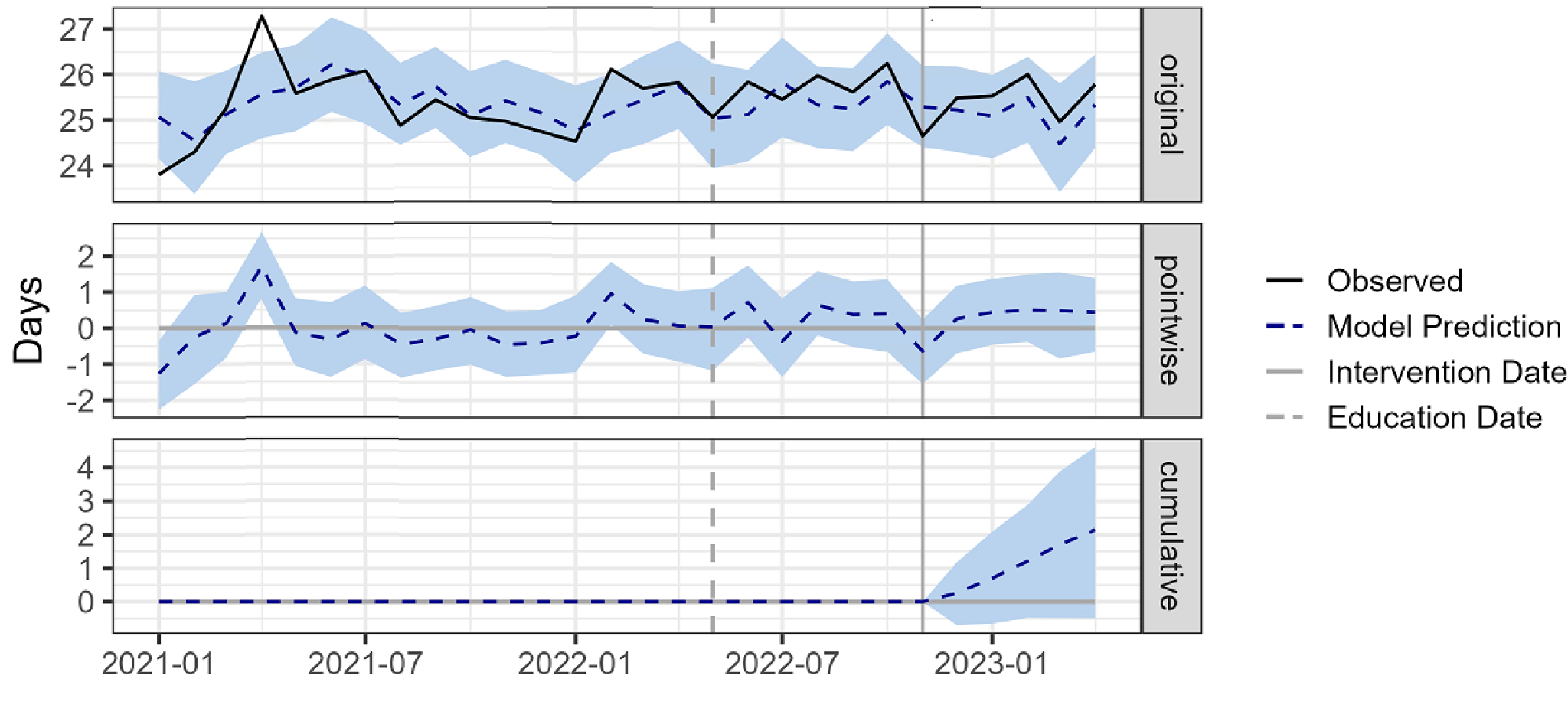


**Supplementary Figure 21. Causal impact analysis of intensive care unit (ICU)-free days.** Plots of the causal impact analysis using a Bayesian structural time-series model. The top subpanel (“original”) shows the actual outcome (black) and the average model predictions (dashed blue) and 95% confidence limits (shaded blue) during the pre-intervention and post-intervention periods, indicated by the solid gray vertical line. The middle subpanel (“pointwise”) shows the difference between the model predictions and the observed outcome. The bottom subpanel (“cumulative”) shows the sum of the pointwise differences during the post-intervention period.

##

## **Supplementary Note 11. Trends of covariates used in causal impact analyses.**

# **
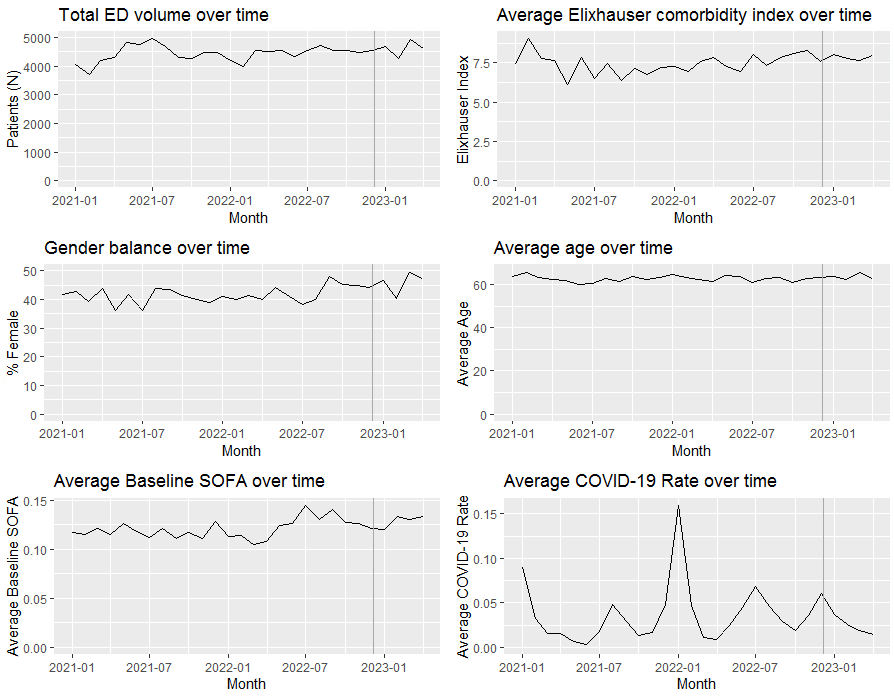
**

**Supplementary Figure 22. Temporal trends of covariates used in the causal impact analyses.** ED = emergency department. SOFA = sequential organ failure assessment score.
